# Role of ultraviolet mutational signature versus tumor mutation burden in predicting response to immunotherapy

**DOI:** 10.1002/1878-0261.12748

**Published:** 2020-07-07

**Authors:** Timothy V. Pham, Amélie Boichard, Aaron Goodman, Paul Riviere, Huwate Yeerna, Pablo Tamayo, Razelle Kurzrock

**Affiliations:** ^1^ Center for Personalized Cancer Therapy Moores Cancer Center UCSD San Diego CA USA; ^2^ Division of Blood and Marrow Transplantation Department of Medicine Moores Cancer Center University of California San Diego La Jolla CA USA; ^3^ Division of Medical Genetics Department of Medicine Moores Cancer Center University of California San Diego La Jolla CA USA; ^4^ Division of Hematology/Oncology Department of Medicine Moores Cancer Center University of California San Diego La Jolla CA USA

**Keywords:** checkpoint blockade, immunotherapy, UV mutational signature

## Abstract

Hydrophobic neoantigens are more immunogenic because they are better presented by the major histocompatibility complex and better recognized by T cells. Tumor cells can evade the immune response by expressing checkpoints such as programmed death ligand 1. Checkpoint blockade reactivates immune recognition and can be effective in diseases such as melanoma, which harbors a high tumor mutational burden (TMB). Cancers presenting low or intermediate TMB can also respond to checkpoint blockade, albeit less frequently, suggesting the need for biological markers predicting response. We calculated the hydrophobicity of neopeptides produced by probabilistic *in silico* simulation of the genomic UV exposure mutational signature. We also computed the hydrophobicity of potential neopeptides and extent of UV exposure based on the UV mutational signature enrichment (UVMSE) score in The Cancer Genome Atlas (TCGA; *N* = 3543 tumors), and in our cohort of 151 immunotherapy‐treated patients. *In silico* simulation showed that UV exposure significantly increased hydrophobicity of neopeptides, especially over multiple mutagenic cycles. There was also a strong correlation (*R* ^2^ = 0.953) between weighted UVMSE and hydrophobicity of neopeptides in TCGA melanoma patients. Importantly, UVMSE was able to predict better response (*P* = 0.0026), progression‐free survival (*P* = 0.036), and overall survival (*P* = 0.052) after immunotherapy in patients with low/intermediate TMB, but not in patients with high TMB. We show that higher UVMSE scores could be a useful predictor of better immunotherapy outcome, especially in patients with low/intermediate TMB, likely due to increased hydrophobicity (and hence immunogenicity) of neopeptides.

## Graphical abstract

The mutational pattern induced by ultraviolet radiation changes the exome to encode for more hydrophobic antigens. When these more lipophilic antigens are presented on the cell surface to T cells by major histocompatibility complex I molecules, they could induce a stronger immune response. This was demonstrated in a cohort of 151 patients from the Moores Cancer Center, particularly in the low TMB subset.

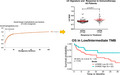

**Abbreviations**
APOBEC: Apolipoprotein B mRNA Editing Enzyme, Catalytic Polypeptide‐like
AU: arbitrary units
CI: confidence interval
CR: complete response
HR: hazard ratio
Mb: megabase
MHC: major histocompatibility complex
MSE: mutational signature enrichment
*N*: number
n.r.: not reached
n.s.: not significant
NGS: next‐generation sequencing
OS: overall survival
PD: partial disease
PD‐1: programmed cell death 1
PD‐L1: programmed death ligand 1
PFS: progression‐free survival
PR: partial response
RECIST: response evaluation criteria in solid tumors
ROC: receiver operating characteristic
SD: stable disease
SKCM: skin cutaneous melanoma
TCGA: The Cancer Genome Atlas
TMB: tumor mutation burden
UV: ultraviolet
UVMSE: UV mutational signature enrichment
VCF: variant call format

## 1 Introduction

Cells in the human body naturally present antigens, which are short peptide fragments derived from intracellular and extracellular sources, on their surfaces in major histocompatibility complex (MHC) proteins. Intracellular antigens are usually presented by MHC class I molecules to effector T cells [1] to help the immune system recognize whether the cell is healthy and whether it belongs to the host (‘self’), whereas extracellular antigens are often displayed in MHC II moieties on professional antigen‐presenting cells like dendritic cells, B cells, and monocytes [2]. Healthy cells displaying valid ‘self’ antigens are not recognized by effector T cells due to negative selection in the thymus,T_reg_ cells meant to suppress the immune system do recognize these cells [2]. In contrast, cancer cells should be recognized and attacked by cytotoxic T cells because malignant cells harbor mutations that manifest as altered peptide neoantigens marking them as nonself. Antigens presented in MHC II could also be important in activating CD4+ ‘helper’ T cells, which are involved in a variety of antitumor responses [2]. Logically, more mutations should result in a greater probability of presenting immunogenic neoantigens on the cell surface [3].

In order to survive and evade the immune response, highly mutated tumor cells use several evasion techniques including downregulating MHC I expression, though natural killer cells are more likely to target these without the involvement of a separate evasion mechanism involving shedding like in some prostate cancers [4], since the presence of MHC I inhibits their activity [2, 5], and expressing immune checkpoint surface proteins, such as programmed cell death 1 (PD‐1) ligands [6] to dull the adaptive response their foreign antigens trigger. PD‐1 ligands are induced by interferon gamma found in the proinflammatory tumor microenvironment [7] and cause CD8+ T cells to become anergic, even if they recognize the foreign antigens present on the tumor. Checkpoint blockade immunotherapies (e.g., anti‐PD‐1/PD‐L1 antibodies) counter this effect by obstructing the PD‐1/PD‐L1 interaction. These are more effective in cancers characterized by a high tumor mutational burden (TMB) such as melanoma [8] and the high TMB subset of patients in other cancers [8, 9, 10, 11, 12, 13, 14]. Other factors that correlate, albeit imperfectly, with a propensity to PD‐1/PD‐L1 inhibitor responsiveness include PD‐L1 overexpression [8, 12, 13] and Apolipoprotein B mRNA Editing Enzyme, Catalytic Polypeptide‐like (APOBEC) mutational activity [15].

Most mutations in melanomas are caused by exposure to ultraviolet (UV) light [16] through the action of free radicals formed by high‐energy UV rays disrupting covalent double bonds in pyrimidine DNA bases [16]. There are three forms of UV radiation, categorized by wavelength, and therefore energy level: UVA (340–400 nm), UVB (280–320 nm), and UVC (200–280 nm). Free radical oxidation reactions due to UVB and UVC light cause dipyrimidine dimers to form, most of which should be repaired by nucleotide excision repair. However, some residual dipyrimidine mutations remain uncorrected, leading to increased brittleness in the DNA helix and improper replication and transcription. Dipyrimidines composed of linked cytosines are usually mispaired with two adenines during DNA replication, resulting in the characteristic CC→TT mutations commonly associated with UV light [17]. Lower energy UVA radiation tends to cause G→T mutations by free radicals oxidizing guanine, creating a new 7,8‐dihydro‐8‐oxoguanine species that can pair with adenine, which then causes the guanine's replacement with a thymine in a succeeding DNA replication cycle [16].

In this paper, we describe biochemical (hydrophobicity) and clinical outcomes as related to UV‐induced hypermutation. We show that neoantigens produced from a UV‐mutated genome tend to be more hydrophobic and therefore are likely to be more immunogenic because they are better presented by MHC and they are more easily recognized by T cells [18, 19, 20]. We also show a positive correlation between response to immunotherapy and level of UV mutations in 151 patients seen at the University of California San Diego Moores Cancer Center. These data suggest that the shift toward hydrophobicity induced by UV mutations is likely to underlie the enhanced responsiveness to immunotherapy.

## 2 Materials and methods

### 2.1 *In silico* UV mutagenesis

We generated all possible 6‐nucleotide stretches (representing two codons) and applied the UV mutational signature, as previously described [21], onto them. The 6‐nucleotide length for each stretch was used to allow us to consider the effect of mutations occurring in all possible reading frames in a codon. This is because the UV mutation signature is defined using the context of the two nucleotides flanking the substitution site since mutations arising from UV exposure often involve reactions between neighboring bases [22], therefore necessitating the presence of at least five nucleotides per stretch to allow the signature to be applied to every possible reading frame of the codon. The 4096 6‐nucleotide stretches, before and after mutation, were then virtually transcribed into their corresponding amino acids whose total hydrophobicity was calculated with the Kyte–Doolittle hydrophobicity scale [23] (with and without the reciprocal strand). The hydrophobicity of the dipeptides was multiplied before and after mutagenesis by the probability of observing the codons corresponding to the dipeptide on the human coding genome (derived from the Kazusa's codon usage database [24]) and the probability of UV mutagenesis on the stretch [21]. The change in hydrophobicity due to *in silico* mutagenesis was compared using the Wilcoxon signed‐rank test.

For example, using the 6‐mer TCCGAG, encoding for the dipeptide Ser‐Glu, would have a Kyte–Doolittle hydrophobicity index of (−0.8) + (−3.5) = (−4.3) arbitrary unit (AU) [23]. This 6‐mer can be mutated with the most frequent UV mutagenesis pattern TCC>TTC at the second position to yield the sequence TCCGAG/Phe‐Glu, which has a hydrophobicity index of (+2.8) + (−3.5) = (−0.7) AU. The substitution alone results in an increase in hydrophobicity of +3.6 AU, but this value must be further weighted by the probability of the mutation occurring in the specific 6‐nucleotide stretch. The mutation occurrence probability is calculated as the joint probability of encountering the original TCCGAG sequence in the genome, 0.00070092, and the probability of the TCC>TTC mutation occurring based on the signature 7 from [21], 0.2887, yielding a joint mutation probability for this specific case of 0.00070092 × 0.2887 = 2.024 × 10^−4^. The relative change in hydrophobicity of this substitution is therefore 2.024 × 10^−4^ × (+3.6) = +72.84 × 10^−4^. Analogous calculations were performed for all possible nucleotide substitutions (three unique nucleotides per position) at all definable, mutable positions (2nd, 3rd, 4th, and 5th positions) in all 6‐nucleotide stretches (*n* = 4096) resulting in a total of 49 152 possible singly mutated stretches, whose relative hydrophobicity changes were summed together to estimate the genome‐wide hydrophobicity change for each round of mutagenesis. Each succeeding cycle of mutagenesis starts with the preceding 6‐nucleotide stretches, and the joint probability of the two codons was modified based on the mutations applied in the previous cycle.

Each iteration of mutagenesis described above corresponds to a single iteration of UV‐mediated mutagenesis, equivalent to an AU dose of UV exposure. Multiple iterations of mutagenesis in this method are intended to correspond to increasing doses of UV light. We repeatedly simulated UV mutagenesis for up to 100 iterations to investigate and model the effects of long‐term UV exposure on antigen hydrophobicity.

### 2.2 Analysis of UV mutational signature in TCGA repository pan‐cancer tumor samples

Molecular profiles, obtained by next‐generation sequencing (NGS) of human tumors, consisting of mutations such as substitutions or small insertions/deletions and mRNA expression data, were downloaded from the community resource project The Cancer Genome Atlas (TCGA), using the Broad GDAC Firehose website (https://gdac.broadinstitute.org—standardized data run release 2016_01_28). All samples were published and available without usage restrictions as of January 14, 2019. All TCGA data used in this study respected the TCGA Human Subjects Protection and Data Access Policies (https://cancergenome.nih.gov/abouttcga/policies/tcga‐human‐subjects‐data‐policies). Another set of mutation data containing mutation data for acral melanomas [25] was downloaded from cBioPortal (http://www.cbioportal.org/study?id=mel_tsam_liang_2017). The acral melanoma data were collected in accordance with the protocol approved by Vanderbilt University and Memorial Sloan‐Kettering Cancer Center Institutional Review Boards, as detailed in Liang *et al*. [25]. The mutation annotation file from both data sources containing the mutation data were then filtered by genomic coordinates corresponding to the exon regions sequenced by Foundation Medicine.

### 2.3 UV mutational signature enrichment estimation for TCGA samples

An estimation of the enrichment of mutations due to UV exposure was performed using our implementation of a signature estimator (publicly available software tool at https://github.com/UCSD‐CCAL/Mutational‐Signature‐Enrichment‐Calculator). The results were given as a numerical score (UV mutational signature enrichment, UVMSE) representing the enrichment of mutations likely to be caused by UV exposure. In TCGA tumors, for each sample the total number of mutations was multiplied by its UVMSE score to quantify the extent of UV‐induced mutagenesis in each sample when correlating it with each sample's overall neopeptide hydrophobicity.

### 2.4 Hydrophobicity analysis

From 9166 samples in the TCGA database (33 distinct tumor types), we selected 3543 tumors without (a) *POLE* and *POLD1* mutations, (b) mismatch repair gene loss, underexpression, or mutations, (c) and microsatellite instability‐high alterations because these alterations are already known to influence immunotherapy response [12, 26, 27]. Using the mutation description available for these tumors, we then performed two types of analysis: (a) In the first analysis, for each tumor, the differences in total hydrophobicity (i.e., the sum of the hydrophobicity of all amino acids) of each transcript’s full‐length peptide product (after versus before mutagenesis) were considered; and (b) for the second analysis, for each tumor, mutated transcripts were used to generate all possible 8‐ to 10‐mer neoantigens encompassing a mutation (since MHC I presents 8–10 amino acid peptides); the differences in total hydrophobicity of the neoantigens after versus before mutagenesis were considered. The results of both (a) and (b) above were computed either not weighted by mRNA expression levels or weighted by these levels (in order to take into consideration whether the neoantigens were actually transcribed and their respective levels of expression). For each sample, the weighted and unweighted hydrophobicities were then correlated against the UVMSE‐weighted total mutation count.

### 2.5 Analysis of UV mutagenesis signature and tumor neoantigen hydrophobicity in patients receiving immunotherapy (PD‐1/PD‐L1 blockade agents)

We reviewed the electronic medical records of 1638 eligible patients with malignancies at UC San Diego Moores Cancer Center who have undergone hybrid capture‐based NGS (Foundation Medicine, Cambridge, MA, USA) starting in October 2012. Only patients having received at least one line of immunotherapy were considered (*N* = 151). For each case, responses to therapy were assessed based on physician notation, using the Response Evaluation Criteria in Solid Tumors (RECIST) criteria. This study was performed in accordance with UCSD Institutional Review Board guidelines for data analysis (NCT02478931) and for any investigational treatments for which patients consented. In addition, the study methodologies conformed to the standards set by the Declaration of Helsinki.

Formalin‐fixed paraffin‐embedded tumor samples from these patients were submitted for NGS to Foundation Medicine’s clinical laboratory improvement amendments‐certified laboratory. The patients’ mutations were assessed with the FoundationOne® assay (hybrid capture‐based panel exome NGS; panel of up to 315 genes—http://www.foundationone.com/). The methods have been previously described in Frampton *et al*. [28]. Average sequencing depth of coverage was greater than 250×, with more 99% of exons covered having greater than 100×. TMB, measured in mutations per megabase (Mb), was calculated by extrapolating the number of somatic mutations detected on NGS to the whole exome with a validated algorithm [26, 29]. Alterations likely or known to be *bona fide* oncogenic drivers and germline polymorphisms were excluded. TMB levels were divided into three groups: low (1–5 mutations/Mb), intermediate (6–19 mutations/Mb), and high (≥ 20 mutations/Mb), which stratified roughly 50% of patients to low TMB, 40% to intermediate TMB, and 10% to high TMB in our cohort [30].

### 2.6 UV mutational signature enrichment (UVMSE) for patient samples

An UVMSE score was computed for each of the 151 patients from their NGS data on the Foundation Medicine gene panel using our mse software tool as was used to calculate the UV MSEs of TCGA data. Demographic data for the patients were previously provided (8).

Variant call format (.VCF) files for the 151 Moores Cancer Center patients were generated by processing the Binary Sequence Alignment/Map format (.BAM) files, obtained from Foundation Medicine Inc. (www.foundationmedicine.com/) NGS, with the variant detection FreeBayes algorithm, and excluding low‐quality variants (QUAL score of < 50 or read depth of < 100). Further, the VCF files were then filtered by genomic coordinates corresponding to the exonic regions Foundation Medicine sequences for their commercially available report. We then defined a single‐strand, DNA‐specific UV signature, signature 7 from [21]. Substitutions in the reverse complement were treated the same as those in the forward strand for counting purposes. The UVMSE score quantifies how frequently mutations described in the defined UV signature 7 occur at a specific sequence context compared to analogous single nucleotide substitutions in other contexts. It is our adaptation of the quantification method described in Roberts *et al*. [31]. For example, the mutation TCC→TTC, the most frequent UV‐induced mutation according to Alexandrov *et al*. [21], can be used to illustrate our method. Its enrichment can be calculated as follows:

(1) $$E_{T\underline{C}C\to T\underline{T}\underline{C}}=\frac{{\text{Mut}}_{T\underline{C}C\to T\underline{T}C}/{\text{Con}}_{T\underline{C}C\to T\underline{T}C}}{{\text{Mut}}_{C}/{\text{Con}}_{C}}=\frac{{\text{Mut}}_{T\underline{C}C\to T\underline{T}C}\times {\text{Con}}_{c}}{{\text{Mut}}_{C}\times {\text{Con}}_{T\underline{C}C\to T\underline{T}C}}$$

Mut_TCC→TTC_ is the amount of TCC→TTC and GGA→GAA reverse complement mutations counted in a 41‐nucleotide stretch on the human genome (GrCh37.75) centered around a detected single nucleotide substitution from the VCF file. Con_TCC→TTC_ is the amount of TCC contexts that can be potentially mutated found in the reference genome copy of the stretch. Mut_C_ is the number of C→T and G→A reverse complement mutations found in the stretch, and Con_C_ is the number of C and G nucleotides found in said stretch. This enrichment value was then weighted by the probability of the mutation occurring, according to the signature [21].

The weighted enrichment value for each of the 192 described mutations in the signature was then summed together to yield the UVMSE score for a particular sample.

### 2.7 Clinical outcome analysis of patients receiving PD‐1/PD‐L1 blockade agents

Patients were divided into two groups of interest: (a) patients presenting a tumor with a high load of UV mutations (‘UV high’); and (b) patients presenting a tumor with a low load of UV mutations (‘UV low’). The optimal threshold (0.7917) for UVMSE estimate was selected using the receiver operating characteristic (ROC) curve method (with UV high being ≥ 0.7917) and evaluating the performance of the UVMSE score to discriminate patient outcomes. (It should be noted that the cutoff in the patient set and the TCGA set for UV high was different, probably because the tumor type distribution differed; for instance, 88 of 3543 (2.5%) of TCGA tumors were melanoma, while 52 of 151 patients (34%) of the patients treated had melanoma, and furthermore, the UV high designation was not used to determine outcome in the TCGA dataset.)

Patients were then grouped by best response: Complete or partial responses (CR/PR) in patients were considered favorable outcomes, whereas a poor outcome was defined as patients with a stable or progressive disease (SD/PD). Best response observed, progression‐free survival (PFS) and overall survival (OS) in months, and TMB and patient demographics were compared between patients presenting a high UVMSE score (≥ 0.7917) versus patients presenting a low UVMSE score.

### 2.8 Statistical analysis and outcome assessment

The association between the UVSME score and clinical outcome was conducted using sas® University Edition software (Cary, NC, USA; http://support.sas.com/software/products/university‐edition/) and graphpad prism® version 6.01 (San Diego, CA, USA; http://www.graphpad.com/scientific‐software/prism/). Two‐tailed tests were used, and *P*‐value ≤ 0.05 was considered significant.

Statistical significance for the *in silico* modeling results was assessed using a Wilcoxon signed‐rank test (nonparametric paired test) for the comparison of change in total hydrophobicity before and after UV mutagenesis in 6‐nucleotide stretches. Change in total hydrophobicity was also calculated in TCGA cohort samples, and those with and without UV mutagenesis were compared; these calculations were performed for the products of full‐length transcripts as well as for 8‐ to 10‐mer peptides; the Mann–Whitney *U*‐test (nonparametric unpaired test) was used.

For the clinical study, Fisher’s exact test was used to assess for the association between categorical variables and the response to therapy, defined as CR/PR, and stable disease or progressive disease (SD/PD) by RECIST criteria. Patient characteristics were summarized using descriptive statistics. Medians and respective 95% confidence intervals (CIs) and range were calculated, whenever possible. The association of UVMSE and TMB levels with PFS and OS in months (calculated using the Kaplan–Meier method) was assessed using the Mantel–Cox log‐rank test. PFS and OS were calculated from the date of starting the immunotherapy. For patients who received multiple immunotherapy regimens, the treatment with the longest PFS was chosen for analysis. Patients were excluded from the survival analysis if they were lost to follow‐up before their first restaging. Patients were censored at date of last follow‐up for PFS or OS, if they had not progressed or died, respectively. In multivariate analysis, associations between categorical variables were tested using a binary logistic regression model and associations between categorical and continuous values such as survival time were assessed with the Cox's proportional hazards model. Linear variables were tested using the Mann–Whitney *U*‐test for univariate analysis.

## 3 Results

**Fig. 1:**
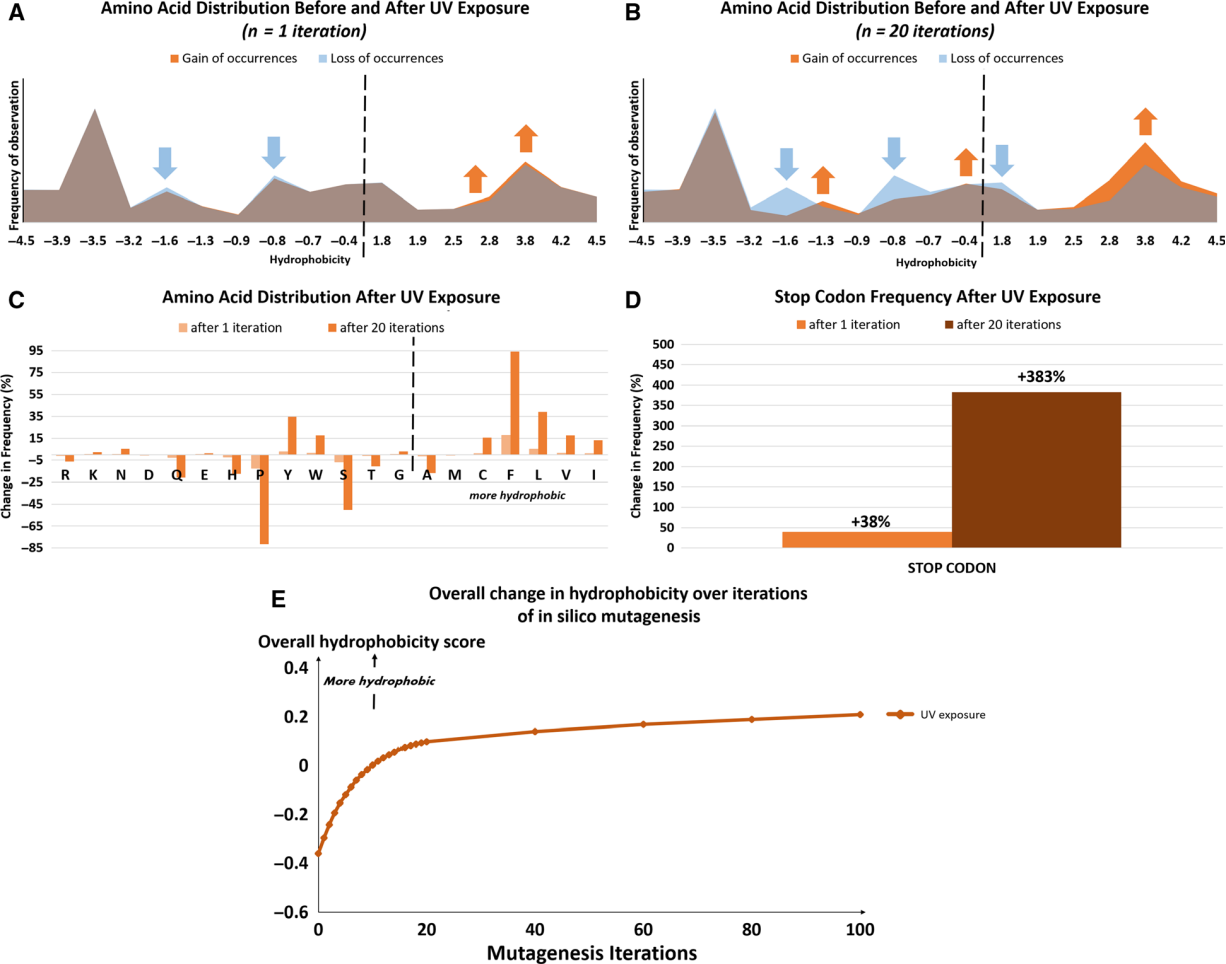
Amino acid distribution and relative hydrophobicity of the human coding genome, after 1 and 20 mutagenesis iterations, as described by *in silico* computation. After 20 *in silico* mutagenesis iterations, the increase in hydrophobicity tabulated in Table 1 becomes even more pronounced. Increasing rounds of mutagenesis cause a loss in hydrophilic amino acid encoding codons and a gain in hydrophobic amino acid encoding codons, therefore increasing the overall hydrophobicity of peptides encoded by the exome, including those of neoantigens. Neoantigen production could potentially increase as well due to an increase in the number of stop codons caused by increasing mutagenesis.

**Fig. 2:**
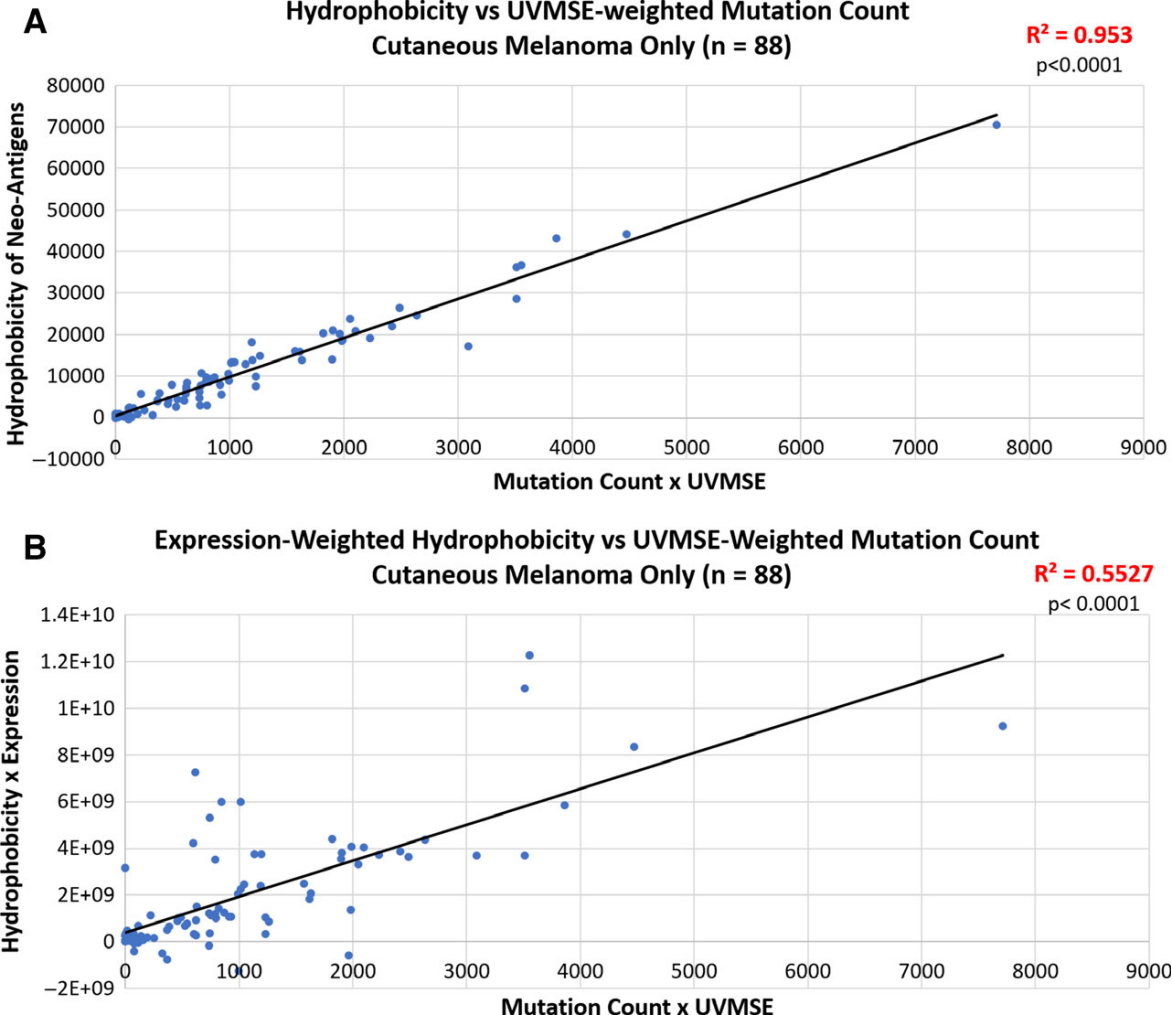
Correlation between the change in hydrophobicity of neoantigens and the total number of mutations in TCGA tumors weighted by UVMSE. In both the pan‐cancer and skin cutaneous melanoma (SKCM) TCGA cohorts, there is a positive correlation between overall neoantigen hydrophobicity and the UVMSE‐weighted mutation count. *P*‐values of the slope were calculated using the standard *t*‐test. Panel A: A significant, positive Spearman correlation of 0.9762 was observed in the 88 SKCM group of the TCGA tumors (*P* < 0.0001). Panel B: When the hydrophobicity was weighted by expression in the same SKCM tumors as panel A, the correlation coefficient remained high at 0.7434 b (*P* < 0.0001).

**Fig. 3:**
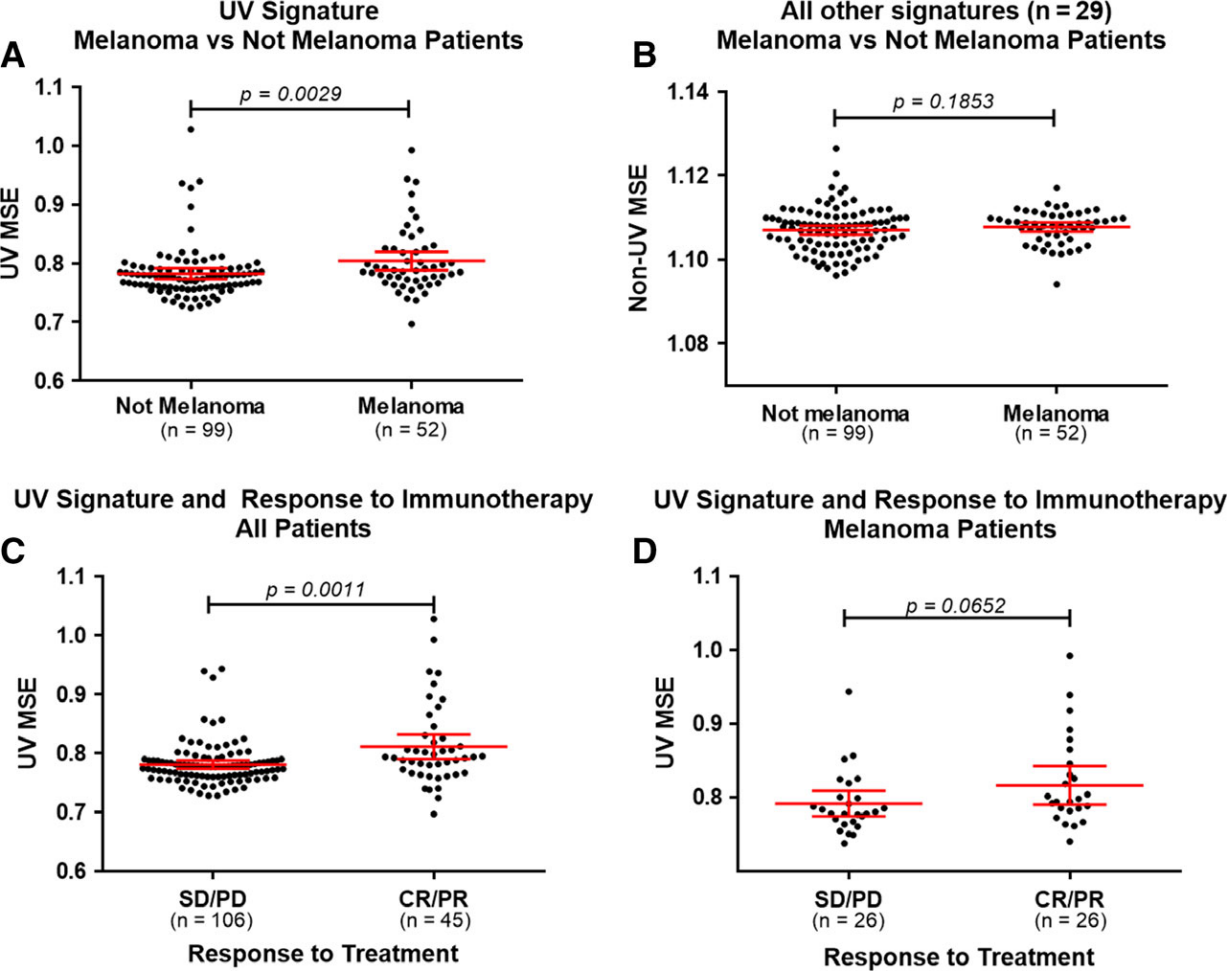
UVMSE analysis in a cohort of 151 patients and correlation with the response to immunotherapy. Patient data were filtered based on genomic coordinates corresponding to the regions sequenced by Foundation Medicine. Red lines indicate mean (95% CI). The UVMSE calculated with only the genomic regions corresponding to those examined by Foundation Medicine for the cohort of 151 Moores Cancer Center patients was used to assess the validity of the UVMSE as a method of measuring degree of UV mutation in tumor samples. See Fig. S1 for the UVMSE calculated on the same cohort with all genomic regions. All *P*‐values were calculated using the Mann–Whitney *U*‐test. UV low < 0.7917 and UV high ≥ 0.7917 (as determined by the UV mutation signature enrichment UVMSE [31]). TMB low = 1–5 mutations/Mb; TMB intermediate = 6–19 mutations/Mb; TMB high ≥ 20 mutations/Mb. Panel A: Comparison of UVMSE in melanoma versus non‐melanoma‐diagnosed patients. Melanoma patients had an average UVMSE of 0.8043 (95% CI: 0.7887–0.8199), while nonmelanoma patients had an average UVMSE of 0.7827 (95% CI: 0.7737–0.7918). The difference was significant with a *P*‐value of 0.0029. Panel B: A comparison of the average of all non‐UVMSE values in melanoma versus non‐melanoma‐diagnosed patients. This difference was n.s. with a *P*‐value of 0.1853, showing that UVMSE is useable as a specific measurement of mutation enrichment due to UV light exposure. Panel C: UVMSE is able to differentiate between negative (SD or PD) and positive (CR or PR) PFS outcomes to immunotherapy in the entire 151‐patient cohort. The average UVMSE of the positive outcome group was 0.7812 (95% CI: 0.7741–0.7882), while the average UVMSE of the negative outcome group was 0.8114 (95% CI: 0.7907–0.8320). This difference was statistically significant with a *P*‐value of 0.0011. Panel D: Within only the cohort of 52 melanoma patients, UVMSE shows a trend in distinguishing between positive and negative outcomes (*P* = 0.0652). The positive outcome group has a higher average UVMSE of 0.8167 (95% CI: 0.7903–0.8430) than the negative outcome group at 0.7919 (95% CI: 0.7746–0.8093; albeit does not reach statistical significance).

**Fig. 4:**
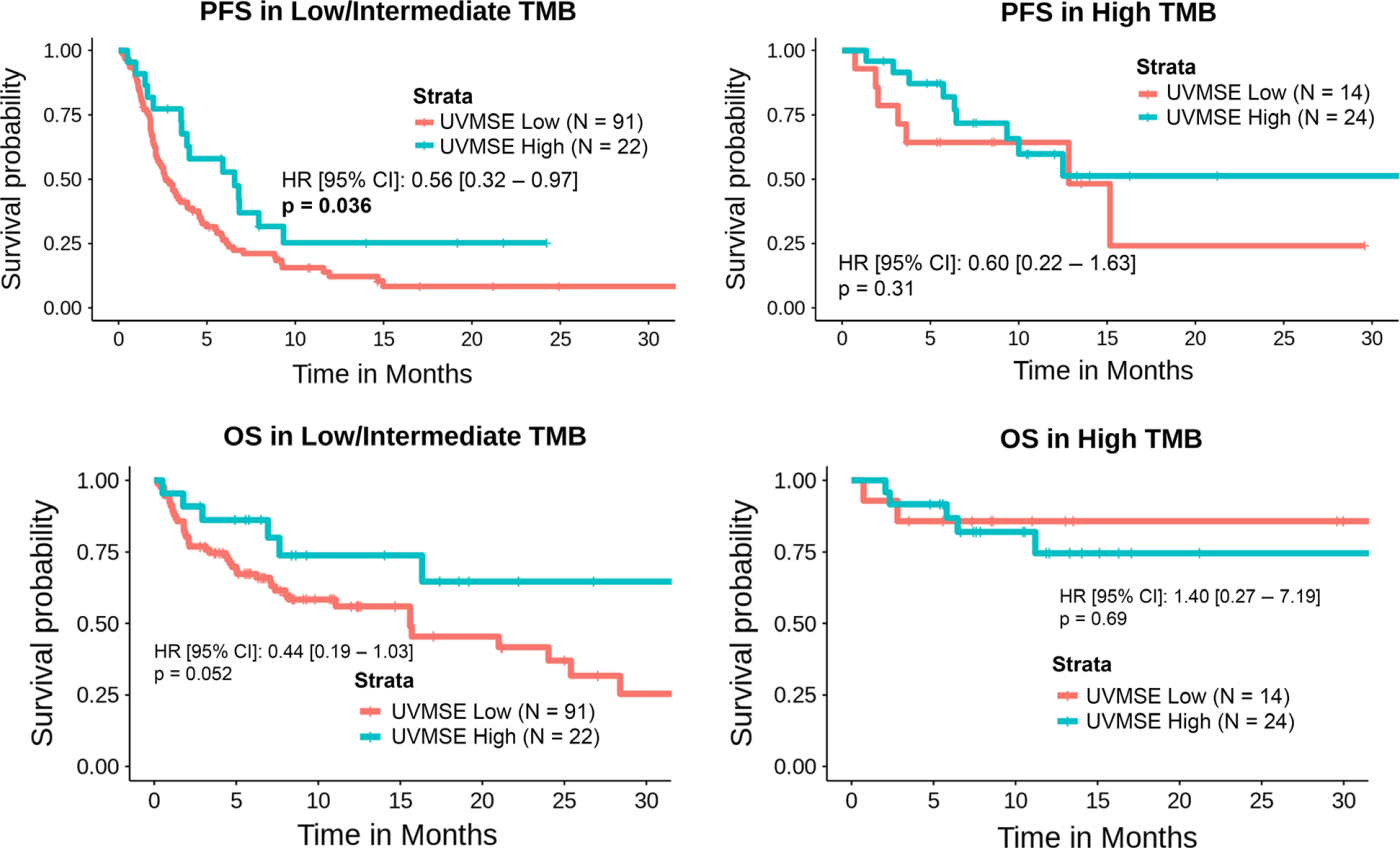
Kaplan–Meier curves of PFS (top panel) and OS (bottom panel) for patients presenting UV signature enrichment compared to those not presenting a UV signature enrichment, in different groups of tumor mutation burden. The figure shows that UVSME level stratifies low/intermediate TMB (but not high TMB) subgroups into those with longer PFS (high UVSME) versus those with shorter PFS (low UVSME; *P* = 0.036) and longer OS (*P* = 0.052). UV low < 0.7917 and UV high ≥ 0.7917 (as determined by the UV mutation signature enrichment UVMSE [31], TMB low = 1–5 mutations/Mb; TMB intermediate = 6–19 mutations/Mb; TMB high ≥ 20 mutations/Mb; All *P*‐values were calculated using the Mantel–Cox log‐rank test..

Overall, both *in silico* and TCGA‐based analysis demonstrated increased hydrophobicity (which is in turn associated with increased immunogenicity) [18, 19, 20] after UV mutagenesis, and that a high UV signature predicts longer PFS and OS in low/intermediate TMB tumors, but not high TMB tumors, after checkpoint blockade (Tables 1, 2 and Tables S1–S9, Figs 1, 2, 3, 4–1, 2, 3, 4 and Figs S1–S2).

**Table 1:** Overall hydrophobicity of the human coding genome increases in a single iteration of UV mutagenesis (per *in silico* computation)*. Table 1 shows the analysis using all existing 6‐nucleotides stretches; alterations on the reciprocal strands were not included because of an existing bias against mutations in the reciprocal strand [21]. See Table S1 for calculations with the use of the reciprocal strand.

	Considering all stretches (one iteration)^a^		
	Hydrophobicity (AU)		
	Before UV mutagenesis	After UV mutagenesis	Difference After–Before UV mutagenesis
Number of stretches	4096		
Median	−0.00003438	−0.00002255	+1.6 × 10^−7^
25th percentile	−0.0006361	−0.0006205	−1.6 × 10^−6^
75th percentile	0.0003892	0.0003933	5.3 × 10^−6^
Mean	−0.0001756	−0.0001695	+5.8 × 10^−6^
Standard deviation	0.001392	0.001391	4.9 × 10^−5^
Standard error	0.00002175	0.00002174	7.7 × 10^−7^
Lower 95% CI	−0.0002182	−0.0002121	4.3 × 10^−6^
Upper 95% CI	−0.0001329	−0.0001269	7.3 × 10^−6^
Sum	−0.7191	−0.6941	**+0.0250**
*P*‐value Wilcoxon signed‐rank test	**< 0.0001**		

Bolded values are meant to highlight statistically significant results.

^a^ UV signature pattern 7 (as described by Alexandrov *et al*. [21] was used. All stretches have at least one mutation. For one iteration, every possible six‐nucleotide combination was generated, and then virtually transcribed to amino acids, and then the hydrophobicity of the amino acids was calculated, and then multiplied by the frequency that the two codons (six nucleotides) would appear in the human genome (probability based on Kazusa’s codon usage database [24]). We then virtually mutated nucleotides 2,3,4, and 5 of each 6 nucleotide stretch (since that would result in all possible configurations for the two codons), and for each mutation, we multiplied the probability that the mutation would occur as part of the UV signature, with the latter being derived from Alexandrov *et al*. [21],finally, the hydrophobicity of the new amino acids would be calculated and multiplied by the probability of the two original codons occurring.

**Table 2:** Patient demographics by UV signature low versus high (*N* = 151)^a^.

Variable	Group	All patients	UV low	UV high	Univariate^b^		Multivariate^c^	
		*N* = 151 (100%)	*N* = 105 (70%)	*N* = 46 (30%)	OR^g^ (95% CI)	*P*‐value	OR^g^ (95% CI)	*P*‐value
Age	≤ 60 years (reference group)	78 (52%)	55 (71%)	23 (29%)	1.1 (0.5–1.8)	0.8602	–	–
	> 60 years	73 (48%)	50 (68%)	23 (32%)				
Gender	Men	93 (62%)	62 (67%)	31 (33%)	1.4 (0.7–3.0)	0.3677	–	–
	Women (reference group)	58 (38%)	43 (74%)	15 (26%)				
Ethnicity	Caucasian	111 (74%)	69 (62%)	42 (38%)	**5.5** (**1.8**–**16.5**)	**0.0011**	**3.5** (**1.1**–**11.1**)	**0.0329**
	Other ethnicities (reference group)	40 (26%)	36 (90%)	4 (10%)				
Tumor type	Melanoma	52 (34%)	28 (54%)	24 (46%)	**3.0** (**1.5**–**6.2**)	**0.0031**	**2.3** (**1.0**–**5.2**)	**0.0427**
	Other tumors^d^ (reference group)	99 (66%)	77 (78%)	22 (22%)				
TMB^e^	High	38 (25%)	14 (37%)	24 (63%)	**7.1** (**3.2**–**15.9**)	**< 0.0001**	**5.6** (**2.4**–**13.2**)	**< 0.0001**
	Low or intermediate (reference group)	113 (75%)	91 (81%)	22 (19%)				
Type of immunotherapy	Anti‐PD‐1/PD‐L1 alone	102 (68%)	77 (75%)	25 (25%)	**0.4** (**0.2**–**0.9**)	**0.0249**	–	0.5888
	Other regimens^f^ (reference group)	49 (32%)	28 (57%)	21 (43%)				
Response	CR/PR	45 (30%)	21 (47%)	24 (53%)	**4.4** (**2.1**–**9.2**)	**0.0002**		
	SD/PD (reference group)	106 (70%)	84 (79%)	22 (21%)				
PFS on immunotherapy (months)	Median (range)	4.6 (0.2–54.7)	3.2 (0.2–54.7)	9.3 (0.5–40.9+)	**HR = 0.4** (**0.3**–**0.7**) **(high versus low UV)**	**0.0001**		
OS from immunotherapy (months)	Median (range)	25.4 (0.2–66.1+)	21 (0.2–66.1+)	n.r. (0.5–51.9+)	**HR = 0.4** (**0.2**–**0.9**) **(high versus low UV)**	**0.0139**		

Bolded values are meant to highlight statistically significant results.

^a^ UV low < 0.7917 and UV high ≥ 0.7917 (as determined by the UVMSE [31]).

^b^ Calculated using Fisher's exact test and log‐rank (Mantel–Cox) test where appropriate.

^c^ Variables presenting a *P*‐value ≤ 0.2 in univariate analysis were included in the multivariate model.

^d^ Tumors included: Adrenal carcinoma (*n* = 1), appendix adenocarcinoma (*n* = 1), basal cell carcinoma (*n* = 2), bladder transitional cell carcinoma (*n* = 4), breast cancer (*n* = 3), cervical cancer (*n* = 2), colorectal adenocarcinoma (*n* = 5), cutaneous squamous cell carcinoma (*n* = 8), hepatocellular carcinoma (*n* = 3), head and neck (*n* = 13), Merkel cell carcinoma (*n* = 2), non‐small‐cell lung carcinoma (*n* = 36), ovarian carcinoma (*n* = 2), pleural mesothelioma (*n* = 1), prostate cancer (*n* = 1), renal cell carcinoma (*n* = 6), sarcoma (*n* = 3), thyroid cancer (*n* = 3), unknown primary squamous cell carcinoma (*n* = 2), and urethral squamous cell carcinoma (*n* = 1)

^e^ TMB low = 1–5 mutations/Mb; TMB intermediate = 6–19 mutations/Mb; TMB high ≥ 20 mutations/Mb. High TMB was compared to low and intermediate TMB.

^f^ Other regimens: OX40 (*n* = 3), anti‐CD73 (*n* = 1), anti‐CTLA4 (*n* = 15), OX40 + anti‐PD‐1 (*n* = 1), anti‐PD‐1 + anti‐CTLA4 (*n* = 17), IDO + anti‐PD‐1 (*n* = 1), high‐dose IL‐2 (*n* = 8), others (*n* = 4).

^g^ OR > 1.0 implies higher chance of response; HR < 1.0 implies less chance of progression or death; and OR and HR refer to UV high versus UV low.

### 3.1 UV mutational signature and increased exome hydrophobicity are associated as determined by *in silico* mutagenesis

Overall, 4096 six‐nucleotide stretches were generated for *in silico* mutagenesis, each consisting of different combinations of the four canonical DNA nucleotides A, T, C, and G (4^6^ = 4096). The 2nd, 3rd, 4th, or 5th position on each stretch was mutated once, separately, per cycle of mutagenesis creating 49 152 possible mutated stretches [4096 stretches × 4 mutable positions × 3 possible mutations (nucleotides other than the preexisting one)]. Overall exome hydrophobicity increased proportionally with the number of *in silico* UV mutagenesis cycles. After one cycle of UV mutagenesis when considering all 4096 possible stretches (*P* < 0.0001), the median hydrophobicity increased by 1.6 × 10^−7^ AU, and the average hydrophobicity increased by 5.8 × 10^−6^ AU (Tables 1 and S1). The calculations were repeated with five nucleotide stretches and showed the same significant increase in hydrophobicity (data not shown).

The number of codons coding for hydrophobic amino acids increased, while the number of hydrophilic amino acid codons decreased with each successive iteration of *in silico* UV mutagenesis (Fig. 1A‐C). The number of stop codons present in the exome also increased with each iteration of mutagenesis: by 38% after one round and by 383% after 20 rounds (Fig. 1D). (Transcripts with premature stop codons tend to become neoantigens due to a quality control mechanism involving the pioneer round of translation in the nucleus [32].) An overall increase in exome hydrophobicity proportional to the number of iterations of *in silico* mutagenesis is the net result of these changes (Fig. 1E).

### 3.2 Neoantigen hydrophobicity correlates with UV exposure mutations in TCGA samples

In the selected TCGA cutaneous melanoma cohort, the total number of mutations in each sample was weighted by the sample’s UVMSE as a proxy for how many UV‐induced mutations are present in the sample. Neoantigen hydrophobicity shows a strong correlation (*R* ^2^ = 0.953, *P* < 0.0001) with UVMSE‐weighted mutation quantity in the selected 88 cutaneous melanoma tumors. Weighting neoantigen hydrophobicity by expression preserves the significance (*R* ^2^ = 0.5527, *P* < 0.0001; Fig. 2).

### 3.3 UVMSE correlates with tumor types that are known to have high UV exposure

Figure S1 shows that melanoma has higher UVMSE versus nonmelanoma (Panel A; *P* = 0.0002), but the MSE of other signatures was not significantly different between the two groups (Panel B) in the 151 UCSD Moores Cancer Center patients. Further, patients who attained a CR or PR after immunotherapy had a higher UVMSE in the pan‐cancer cohort (*P* = 0.0165), but this difference in UVMSE was not significant (n.s.) in the melanoma patients, perhaps because of the small number of cases (Panels C and D).

The UVMSE scores of the cutaneous melanoma cohort in TCGA were also compared with those from the acral melanoma cohort [25] in order to determine whether UVMSE can accurately predict high or low UV exposure (Fig. S2). The acral samples were all placed in the ‘low UV’ group because of the low rate of UV mutation in acral melanomas [25]. The low UV group had an average UVMSE of 1.206 (95% CI: 0.8290–1.583), while the high UV group had an average UVMSE of 1.842 (95% CI: 1.762–1.922; calculated on whole exome). The difference between the two groups was statistically significant (*P* < 0.0001). We therefore used the data to determine a threshold of 1.642 using the ROC curve method to dichotomize the TCGA data between low and high UV exposure. This threshold has a sensitivity of 73.10% and a specificity of 73.68% when used to predict whether a TCGA patient was diagnosed with cutaneous melanoma.

In our cohort of 151 patients, the mean UVMSE for patients with a melanoma diagnosis, which we used as a proxy for high UV exposure status due to the most common etiology of melanoma being excessive UV exposure [16], was significantly higher at 0.8043 (95% CI: 0.7887–0.8199) compared to nonmelanoma patients who had an average UVMSE of 0.7827 (95% CI: 0.7737–0.7918; *P* = 0.0029; calculated on the genomic real estate in the Foundation Medicine panel; Fig. 3).

### 3.4 Clinical factors associated with high UV signature

In a univariate model based on our 151 patients in the UCSD Moores Cancer Center Cohort, the factors associated with high UV signature included Caucasian ethnicity, tumor type being melanoma, and TMB high (all *P* < 0.003; Table 2).

### 3.5 Univariate and multivariate analyses of factors associated with response, PFS, and OS in immunotherapy‐treated patients

Overall, 151 patients from the UCSD Moores Cancer Center were analyzed for immunotherapy response. Fifty‐two percent were ≤ 60 years old; 62% were men; 74% were Caucasian; 34% had melanoma; 25% had high TMB; and 68% received a PD1/PDL1 inhibitor as a single agent (Table 2). Overall, 30% of patients achieved a CR or PR. The median PFS for all patients was 4.6 months, and median OS was 25.4 months (Table 2).

#### 3.5.1 Univariate analysis of all patients

A UVMSE threshold value of 0.7917 was designated to distinguish between UV‐high and UV‐low status in patients (threshold determined using the ROC curve method; e.g., we identify the threshold that maximizes the area under the ROC for predicting clinical outcome using the UVSME as predictor).

Table S8 shows that tumor type melanoma, TMB high, UV high, and immunotherapies other than single‐agent checkpoint inhibitors were significantly associated with better response rates as well as longer PFS and OS (all *P* < 0.01; univariate analysis). Overall, 24/46 patients (52% of UVMSE high) versus 21/105 (20% of UVMSE low) patients responded (*P* = 0.0002; Table S8). In the UV‐high group, the median PFS was 9.3 versus 3.2 months (*P* = 0.0001) in the UV‐low group, whereas median OS was not reached (n.r.) in the UV‐high group compared to 21 months in the UV‐low group (*P* = 0.0139; Table S8).

#### 3.5.2 Multivariate analysis of all patients

Multivariate analysis demonstrated that only melanoma and TMB high were selected as independent variables predicting response rate, PFS, and OS (all *P* < 0.03; Table S8).

Results were similar in that UV high versus UV low was associated with response in univariate but not multivariate analysis when only nonmelanoma or only melanoma patients were analyzed (Tables S2 and S3). For PFS, UV high was selected as an independent factor predicting PFS only in melanoma (but not in nonmelanoma) patients (Tables S4 and S5). OS could not be associated with any factor once the groups were split into nonmelanoma and melanoma patients, perhaps because of the limited number of patients in each group (Tables S6 and S7).

### 3.6 UV high versus UV low predicts favorable outcome in the TMB‐low/intermediate but not the TMB‐high subgroup

Considering the lower and higher TMB groups separately, UVMSE score was effective at identifying responders in the low/intermediate TMB group (*P* = 0.0026) where 10/22 (45%) patients with a high UVMSE responded to immunotherapy versus 13/91 (14%) of patients with a low UVMSE [odds ratio (OR) of 5.0 (1.8–13.9), *P* = 0.0026]. The results in the high TMB group were n.s. (*P* = 1.0000), with 14/24 (58%) of UV‐high patients responding to treatment versus 8/14 (57%) of UV‐low patients [OR of 1.1 (0.3–4.0); Table S9]. Similar results were seen for PFS and OS: In the low/intermediate TMB group, the UV‐high and UV‐low status predicted longer PFS (*P* = 0.036) and OS (*P* = 0.052) but did not stratify PFS or OS for the TMB‐high patients (Fig. 4).

There was a significant association between UVMSE and clinical response in univariate (*P* = 0.0026) and in multivariate (*P* = 0.0108) analysis among patients with low or intermediate TMB. Within those patients, melanoma tumor type was also significant in both univariate (*P* = 0.0041) and multivariate analyses (*P* = 0.0139), but immunotherapy type was only significant in univariate analysis (*P* = 0.0101; Table S10). UVMSE was significantly associated with PFS or OS time in univariate analysis (*P* = 0.036 and *P* = 0.05) within the low TMB group, but not in multivariate analysis (Fig. 4 and Tables S11 and S12); since only 22 patients were in the high UVSME group in this subanalysis, the small numbers of patients may have precluded robust correlations.

## 4 Discussion

Immunotherapy such as checkpoint blockade has been lauded in both the scientific and popular press because it can effectively suppress or even eradicate some advanced cancers. This phenomenon is due to the way immunotherapy serves as a force multiplier for the body’s endogenous immune system by reactivating it so that cancer cells are recognized and attacked [33]. While immunotherapy results in exceptional responses in certain tumors, such as melanoma, which is characterized by a high TMB [8], it only has a ~ 20% overall response rate in the unselected population of patients with malignancies [8]; further, in certain cases, checkpoint blockade may result in hyperprogression of the tumor and it is not without toxicity [34]. Interestingly, other factors such as *PD‐L1* amplification may also predict immunotherapy response [35]. It is apparent that more predictive biomarkers in addition to histologic diagnosis, TMB, and a better understanding of immunosuppressive mechanisms that incapacitate the natural immune response are needed to more effectively route the appropriate therapies to patients.

We show that the mutational landscape caused by UV light, as quantified by the UVMSE, is positively correlated with increased hydrophobicity of exome protein products in both *in silico* simulation and pan‐cancer TCGA data. This observation is similar to that for APOBEC signatures, which also increase hydrophobicity, but differs from other signatures such as microsatellite instability and tobacco, which may decrease hydrophobicity (even while increasing number of mutations) [15]. The increased hydrophobicity of UV‐mutated proteins derived from the altered coding genome would increase the immunogenicity of the antigens derived from them because T cells have a higher affinity for more hydrophobic antigen peptides [18] and because more hydrophobic peptides bind more strongly to MHC class I molecules’ hypervariable regions, particularly when the antigen’s hydrophobic peptides are located at the anchor positions [19, 36]. An increased number of UV mutations, which bias the resulting peptides toward increased hydrophobicity, would logically increase the probability of hydrophobic peptides being placed at the anchor position. In addition, antigens intended for presentation in MHC I are only 8–10 amino acids long [2], with one or two of those being anchor peptides, so changing only the anchor positions may have important effects. Similar effects regarding antigen hydrophobicity enhancing presentation in MHC II have also been noted since the peptide binding groove of MHC II requires hydrophobic amino acids at key locations [37, 38]. However, antigens bound to MHC II can be larger than those in MHC I due to the open structure of MHC II, and antigens with more hydrophilic external peptides have been shown to be more immunogenic to CD4+ T cells interacting with MHC II [39]. The fact that the number of UV mutations is associated with an increase in putative neoantigen hydrophobicity is a possible explanation for why melanomas tend to respond well to checkpoint blockade immunotherapy [40]. In addition, the increase in the number of stop codons also increases the number of neoantigens produced from the mutated exome due to the resulting excess of faulty transcription products being routed to and processed into antigens via mechanisms such as nonsense‐mediated decay and a separate quality control process located in the nucleus that is part of the pioneer round of transcription [32].

In our study, higher UVMSEs correlated with response to therapy, PFS, and OS. However, despite its significance in univariate analysis, we found that the UV mutational signature was not an independent variable predicting outcome in multivariate analysis; the tumor type, specifically a melanoma versus nonmelanoma histology, and TMB (high versus low/intermediate) were independent predictors of better outcome. These observations are consistent with UV exposure being strongly associated with melanoma diagnoses due to it being the predominant etiology for the disease [16]. Even so, UVMSE can effectively detect responders as well as those with longer PFS and OS after immunotherapy in the low or intermediate TMB patient cohort. This could be explained by the extremely hydrophobic nature (hydrophobicity being associated with immunogenicity [18]) of the UV mutational signature, hence driving the immune response. On the other hand, UVMSE does not appear to be predictive of better outcome in the high TMB cohort, possibly because the sheer number of neoantigens arising from a highly mutated sample, regardless of mutational source, would elicit a strong immune response.

There are several limitations to this study. For instance, the range of UVMSE signatures differs when the patient cancer type distribution differs. This issue can be seen in the range of the UVMSE in TCGA compared to that of the 151 Moores Cancer Center patients. Second, patients had a variety of tumor types and immunotherapies, and the limited number of patients with individual tumor types precluded an analysis by histology. However, the data may also suggest that the results are generalizable across cancers and treatments.

## 5 Conclusion

In summary, we show, through *in silico* simulation and analysis of TCGA data, that a genome altered with the characteristic UV exposure mutation signature would produce 8–10 mer antigens of significantly elevated hydrophobicity. The hydrophobicity of these neoantigens is also proportional to the number of mutations caused by UV exposure in individual samples. This increased hydrophobicity, among other physicochemical properties, may cause T cells of the immune system to recognize cells presenting the neoantigens extracellularly in MHC I molecules as ‘foreign’, marking these cancer cells for destruction. T cells preferentially bind through their T‐cell receptors to more hydrophobic antigens, both because these antigens are more likely to be presented in MHC I moieties due to the requirement for hydrophobic anchor positions to facilitate antigen presentation, a prerequisite for interacting with T cells, and because of the hydrophobic peptides’ intrinsically immunogenic nature [15, 18, 19, 20]. Therefore, checkpoint blockade immunotherapies are more likely to be effective since the tumor will be infiltrated by T cells attached to cancer cells prevented from doing their effector functions only by PD‐1/PD‐L1 interactions. The correlation of UV exposure with better immunotherapy outcome appears to be more important in cases with low/intermediate TMB (versus high TMB), perhaps because, in the latter, the large number of mutations already permits immune recognition once T cells are reactivated after checkpoint blockade therapy.

## Conflict of interest

Kurzrock has research funding from Genentech, Merck Serono, Incyte, Pfizer, Sequenom, Foundation Medicine, Konica Minolta, Grifols, and Guardant Health, as well as consultant fees from XBiotech, Loxo, NeoMed, Gaido, and Actuate Therapeutics, speaker and consultant fees from Roche, and has an equity interest in IDbyDNA and in CureMatch, Inc. Goodman receives speaker fees from Seattle Genetics and consulting fees from Jazz Pharmaceuticals. PR is a paid employee of Peptide Logic, LLC.

## Author contributions

TP and AB performed most of the data analyses. TP and AB performed iterative *in silico* mutagenesis simulation. AG provided and analyzed clinical outcome data on the 151 patients. AB and PR performed statistical analysis on clinical data. TP, HY, and PT developed UVMSE calculating tool. RK provided scientific advice and administrative support. All authors reviewed the manuscript.

## Supporting information


**Table S1**. Consequences of a single iteration of UV mutagenesis on the overall hydrophobicity of the human coding genome, including mutations appearing on the reciprocal strand (computed *in silico*)*.
**Table S2**. Univariate and multivariate analysis of factors affecting response rate for non‐melanoma patients treated with immunotherapy agents (*N* = 99).
**Table S3**. Univariate and multivariate analysis of factors affecting response rate for melanoma patients treated with immunotherapy agents (*N* = 52).
**Table S4**. Factors associated with PFS on immunotherapy for 99 non‐melanoma patients treated with immunotherapy.
**Table S5**. Factors associated with PFS on immunotherapy for 52 melanoma patients treated with immunotherapy.
**Table S6**. Factors associated with OS on immunotherapy for 99 non‐melanoma patients treated with immunotherapy.
**Table S7**. Factors associated with OS on immunotherapy for 52 melanoma patients treated with immunotherapy.
**Table S8**. Univariate and multivariate analysis of factors affecting response rate, progression‐free and overall survival for all patients treated with immunotherapy agents (*N* = 151).
**Table S9**. Factors associated with response to immunotherapy for the treated 151 patients separated into higher and lower TMB groups.
**Table S10**. Univariate and multivariate analysis of factors affecting response rate for low/intermediate TMB patients treated with immunotherapy agents (*N* = 113).
**Table S11**. Factors associated with PFS on immunotherapy for 113 low/intermediate TMB patients treated with immunotherapy.
**Table S12**. Factors associated with OS on immunotherapy for 113 low/intermediate TMB patients treated with immunotherapy.
**Fig**.** S1**. UV signature enrichment analysis in a cohort of 151 patients and correlation to the response to immunotherapy.
**Fig**.** S2**. UV signature enrichment analysis in a cohort of 328 acral and cutaneous melanomas.
