# Supplementary material for: Role of ultraviolet mutational signature versus tumor mutation burden in predicting response to immunotherapy
Source: Mol Oncol. 2020 Jul 7;14(8):1680–94. doi: 10.1002/1878-0261.12748 (PMC7400787; doi:10.1002/1878-0261.12748)
Supplement: Supplementary file 1 — Table S1. Consequences of a single iteration of UV mutagenesis on the overall hydrophobicity of the human coding genome, including mutations appearing on the reciprocal strand (computed in silico)*. Table S2. Univariate and multivariate analysis of factors affecting response rate for non‐melanoma patients treated with immunotherapy agents (N = 99). Table S3. Univariate and multivariate analysis of factors affecting response rate for melanoma patients treated with immunotherapy agents (N = 52). Table S4. Factors associated with PFS on immunotherapy for 99 non‐melanoma patients treated with immunotherapy. Table S5. Factors associated with PFS on immunotherapy for 52 melanoma patients treated with immunotherapy. Table S6. Factors associated with OS on immunotherapy for 99 non‐melanoma patients treated with immunotherapy. Table S7. Factors associated with OS on immunotherapy for 52 melanoma patients treated with immunotherapy. Table S8. Univariate and multivariate analysis of factors affecting response rate, progression‐free and overall survival for all patients treated with immunotherapy agents (N = 151). Table S9. Factors associated with response to immunotherapy for the treated 151 patients separated into higher and lower TMB groups. Table S10. Univariate and multivariate analysis of factors affecting response rate for low/intermediate TMB patients treated with immunotherapy agents (N = 113). Table S11. Factors associated with PFS on immunotherapy for 113 low/intermediate TMB patients treated with immunotherapy. Table S12. Factors associated with OS on immunotherapy for 113 low/intermediate TMB patients treated with immunotherapy. Fig. S1. UV signature enrichment analysis in a cohort of 151 patients and correlation to the response to immunotherapy. Fig. S2. UV signature enrichment analysis in a cohort of 328 acral and cutaneous melanomas. [file MOL2-14-1680-s001.docx]

## Supplemental Table 1: Consequences of a single iteration of UV mutagenesis on the overall hydrophobicity of the human coding genome, including mutations appearing on the reciprocal strand (computed *in silico*)*

The table shows that the overall hydrophobicity of the human coding genome increases in a single iteration of UV mutagenesis even when considering the reciprocal strand. The Mann-Whitney U-test was used to calculate the p-value.

|  | **Considering all stretches (one iteration)*** | | |
| --- | --- | --- | --- |
|  | **HYDROPHOBICITY (AU)** | | |
|  | **Before**  **UV Mutagenesis** | **After**  **UV Mutagenesis** | **Difference**  **After-Before**  **UV Mutagenesis** |
| **Number of stretches** | 4096 | | |
| **Median** | -0.00003438 | -0.00002042 | +1.8 x 10^-7^ |
| 25% percentile | -0.0006361 | -0.0006262 | -3.0 x 10^-6^ |
| 75% percentile | 0.0003892 | 0.0003932 | 9.2 x 10^-6^ |
| **Mean** | -0.0001756 | -0.0001724 | +2.9 x 10^-6^ |
| Standard deviation | 0.001392 | 0.001393 | 3.4 x 10-5 |
| Standard error | 0.00002175 | 0.00002177 | 5.4 x 10^-7^ |
| Lower 95% CI | -0.0002182 | -0.0002150 | 1.9 x 10^-6^ |
| Upper 95% CI | -0.0001329 | -0.0001297 | 4.0 x 10^-6^ |
| **Sum** | -0.7191 | -0.7060 | **+ 0.0131** |
| **P-value**  Wilcoxon signed rank test | **<0.0001** | | |

***** UV signature pattern 7 (as described by Alexandrov et al(Alexandrov *et al.*, 2013)) was used.

Alterations on the reciprocal strands were included, and all existing 6-nucleotides stretches were used. When the reciprocal strand was included, all 6-nucleotide stretches presented at least one mutation. See **Table 1** for data excluding the reciprocal strands.

**Abbreviations:** CI = confidence interval; UV = ultraviolet..

**Supplemental Table 2: Univariate and multivariate analysis of factors affecting response rate for non-melanoma patients treated with immunotherapy agents (N = 99)**

In non-melanoma patients treated with immunotherapy, UVMSE was significantly associated with clinical response in univariate analysis (p = 0.0309) but not in multivariate analysis. TMB is significant in both univariate (p = 0.0027) and multivariate (p = 0.0021) analysis.

| **Variable** | **Group** | **Non-melanoma patients** | **CR/PR** | **SD/PD** | **Univariate^1^** | | **Multivariate^2^** | |
| --- | --- | --- | --- | --- | --- | --- | --- | --- |
|  |  | **N = 99 (100%)** | **N = 19 (19%)** | **N = 80 (81%)** | **Odds Ratio**  **[95% CI]** | **P-value** | **Odds Ratio [95% CI]** | **P-value** |
| **Age** | ≤60 years (reference group) | 47 (47%) | 7 (15%) | 40 (85%) | 0.6 [0.2-1.6] | 0.3217 | - | - |
|  | >60 years | 52 (53%) | 12 (23%) | 40 (77%) |  |  |  |  |
| **Gender** | Men | 58 (59%) | 15 (26%) | 43 (74%) | 3.2 [1.0-10.6] | 0.0683 | - | 0.1198 |
|  | Women (reference group) | 41 (41%) | 4 (10%) | 37 (90%) |  |  |  |  |
| **Ethnicity** | Caucasian | 67 (68%) | 15 (22%) | 52 (78%) | 2.0 [0.6-6.7] | 0.2873 | - | - |
|  | Other ethnicities (reference group) | 32 (32%) | 4 (13%) | 28 (88%) |  |  |  |  |
| **TMB^3^** | High | 20 (20%) | 9 (45%) | 11 (55%) | **5.6 [1.9-17.0]** | **0.0027** | **5.6 [1.9-17.0]** | **0.0021** |
|  | Low or intermediate (reference group) | 79 (80%) | 10 (13%) | 69 (87%) |  |  |  |  |
| **Type of immunotherapy** | Anti-PD-1/PD-L1 alone | 91 (92%) | 16 (18%) | 75 (82%) | 0.4 [0.1-1.6] | 0.1780 | - | 0.2223 |
|  | Other regimens (reference group) | 8 (8%) | 3 (38%) | 5 (63%) |  |  |  |  |
| **UV^4^** | High | 22 (22%) | 8 (36%) | 14 (64%) | **3.4 [1.2-10.1]** | **0.0309** | - | 0.1886 |
|  | Low (reference group) | 77 (78%) | 11 (14%) | 66 (86%) |  |  |  |  |

^1^Calculated using Fisher's exact test.

^2^Variables presenting a p-value ≤0.2 in univariate analysis were included in the multivariate model.

^3^TMB low = 1- 5 mutations/megabase; TMB intermediate = 6-19 mutations/megabase; TMB high ≥ 20 mutations/megabase

^4^UV low < 0.7917 and UV high ≥ 0.7917 (as determined by the UV mutation signature enrichment UVMSE (Roberts *et al.*, 2013)).

**Abbreviations**: 95% CI = 95% confidence interval; CR = complete response; N = number; n.s. = non-significant; PD-1 = programmed death receptor-1; PD-L1 programmed death receptor-ligand 1; PD = progressive disease: PR = partial response; SD = stable disease; TMB = tumor mutational burden; UV = ultraviolet; UVMSE = UV Mutational Signature Enrichment.

## Supplemental Table 3: Univariate and multivariate analysis of factors affecting response rate for melanoma patients treated with immunotherapy agents (N = 52)

In melanoma patients treated with immunotherapy, UVMSE was associated with clinical response in univariate analysis but not in multivariate analysis. TMB is significant in both univariate (p = 0.0399) and multivariate analysis (p = 0.0235).

| **Variable** | **Group** | **Melanoma patients** | **CR/PR** | **SD/PD** | **Univariate^1^** | | **Multivariate^2^** | |
| --- | --- | --- | --- | --- | --- | --- | --- | --- |
|  |  | **N = 52 (100%)** | **N = 26 50%)** | **N = 26 (50%)** | **Odds Ratio**  **[95% CI]** | **P-value** | **Odds-ratio [95% CI]** | **P-value** |
| **Age** | ≤60 years (reference group) | 31 (60%) | 13 (42%) | 18 (58%) | 0.4 [0.1-1.4] | 0.2581 | - | - |
|  | >60 years | 21 (40%) | 13 (62%) | 8 (38%) |  |  |  |  |
| **Gender** | Men | 35 (67%) | 18 (51%) | 17 (49%) | 1.2 [0.4-3.8] | 1.0000 | - | - |
|  | Women (reference group) | 17 (33%) | 8 (47%) | 9 (53%) |  |  |  |  |
| **Ethnicity** | Caucasian | 44 (85%) | 23 (52%) | 21 (48%) | 1.8 [0.4-8.6] | 0.7030 | - | - |
|  | Other ethnicities (reference group) | 8 (15%) | 3 (38%) | 5 (63%) |  |  |  |  |
| **TMB^3^** | High | 18 (35%) | 13 (72%) | 5 (28%) | **4.2 [1.2-14.5]** | **0.0399** | **4.2 [1.2-14.5]** | **0.0235** |
|  | Low or intermediate (reference group) | 34 (65%) | 13 (38%) | 21 (62%) |  |  |  |  |
| **Type of immunotherapy** | Anti-PD-1/PD-L1 alone | 11 (21%) | 5 (45%) | 6 (55%) | 0.8 [0.2-3] | 1.0000 | - | - |
|  | Other regimens (reference group) | 41 (79%) | 21 (51%) | 20 (49%) |  |  |  |  |
| **UV^4^** | High | 24 (46%) | 16 (67%) | 8 (33%) | **3.6 [1.1-11.3]** | 0.0505 | - | 0.1320 |
|  | Low (reference group) | 28 (54%) | 10 (36%) | 18 (64%) |  |  |  |  |

^1^Calculated using Fisher's exact test.

^2^Variables presenting a p-value ≤0.2 in univariate analysis were included in the multivariate model.

^3^TMB low = 1- 5 mutations/megabase; TMB intermediate = 6-19 mutations/megabase; TMB high ≥ 20 mutations/megabase

^4^UV low < 0.7917 and UV high ≥ 0.7917 (as determined by the UV mutation signature enrichment UVMSE (Roberts *et al.*, 2013)).

**Abbreviations**: 95% CI = 95% confidence interval; CR = complete response; N = number; n.s. = non-significant; PD-1 = programmed death receptor-1; PD-L1 programmed death receptor-ligand 1; PD = progressive disease: PR = partial response; SD = stable disease; TMB = tumor mutational burden; UV = ultraviolet; UVMSE = UV Mutational Signature Enrichment.

## Supplemental Table 4: Factors associated with PFS on immunotherapy for 99 non-melanoma patients treated with immunotherapy

UVMSE is an independent factor predicting PFS in non-melanoma patients treated with immunotherapy in univariate analysis (p = 0.0392). However, it is not significant in multivariate analysis. TMB is significant in both univariate (p = 0.0002) and multivariate (p = 0.0002) analysis.

| **Variable** | **Group** | **Non-melanoma patients** | **PFS (months)** | **Univariate^1^** | | **Multivariate^2^** | |
| --- | --- | --- | --- | --- | --- | --- | --- |
|  |  | **N = 99 (100%)** | **Median (range)** | **Hazard Ratio**  **[95% CI]** | **P-value** | **Hazard Ratio**  **[95% CI]** | **P-value** |
| **Age** | ≤60 years | 47 (47%) | 3.2 (2.0-3.9) | - | 0.2179 | - | - |
|  | >60 years | 52 (53%) | 2.7 (1.8-5.9) |  |  |  |  |
| **Gender** | Men | 58 (59%) | 3.1 (1.8-3.9) | - | 0.0924 | - | 0.2246 |
|  | Women | 41 (41%) | 2.9 (2.0-5.9) |  |  |  |  |
| **Ethnicity** | Caucasian | 67 (68%) | 3.5 (1.9-5.9) | - | 0.2183 | - | - |
|  | Other ethnicities | 32 (32%) | 2.3 (1.8-3.5) |  |  |  |  |
| **TMB^3^** | High | 20 (92%) | 10.0 (3.2-nr) | **0.3 [0.2-0.6]** | **0.0002** | **0.3 [0.2-0.6]** | **0.0002** |
|  | Low or intermediate | 79 (92%) | 2.1 (1.8-3.1) |  |  |  |  |
| **Type of immunotherapy** | Anti-PD-1/PD-L1 alone | 91 (92%) | 2.9 (2.0-3.8) | - | 0.8659 | - | - |
|  | Other immunotherapy regimens^4^ | 8 (8%) | 2.6 (1.3-nr) |  |  |  |  |
| **UV^5^** | High | 22 (22%) | 5.9 (2.9-10.0) | **0.6 [0.3-1.0]** | **0.0392** | - | 0.7627 |
|  | Low | 77 (78%) | 2.1 (1.9-3.2) |  |  |  |  |

^1^Calculated using the log-rank test.

^2^Variables presenting a p-value ≤0.2 in univariate analysis were included in the multivariate model.

^3^TMB low = 1- 5 mutations/megabase; TMB intermediate = 6-19 mutations/megabase; TMB high ≥ 20 mutations/megabase

^4^Other immunotherapy: OX40 (n=3), anti-CD73 (n=1), anti-CTLA4 (n=15), OX40+anti-PD-1 (n=1), anti-PD-1+anti-CTLA4 (n=17), IDO+anti-PD-1 (n=1), high dose IL-2 (n=8), others (n=4).

^5^UV low < 0.7917 and UV high ≥ 0.7917 (as determined by the UV mutation signature enrichment UVMSE (Roberts *et al.*, 2013)).

**Abbreviations**: 95% CI = 95% confidence interval; N = number; n.s. = non-significant; nr = not reached; PFS = progression-free survival; PD-1 = programmed death receptor-1; PD-L1 programmed death receptor-ligand 1; TMB = tumor mutational burden; UV = ultraviolet.

## Supplemental Table 5: Factors associated with PFS on immunotherapy for 52 melanoma patients treated with immunotherapy

UVMSE is an independent factor predicting PFS in melanoma patients treated with immunotherapy in both univariate (p = 0.0329) and multivariate (p = 0.0389) analysis.

| **Variable** | **Group** | **Melanoma patients** | **PFS (months)** | **Univariate^1^** | | **Multivariate^2^** | |
| --- | --- | --- | --- | --- | --- | --- | --- |
|  |  | **N = 52 (100%)** | **Median (range)** | **Hazard Ratio**  **[95% CI]** | **P-value** | **Hazard Ratio**  **[95% CI]** | **P-value** |
| **Age** | ≤60 years | 31 (60%) | 8.9 (5.5-54.7) | - | 0.5721 | - | - |
|  | >60 years | 21 (40%) | 12.8 (5.7-nr) |  |  |  |  |
| **Gender** | Men | 35 (67%) | 9.3 (5.8-54.7) | - | 0.6618 | - | - |
|  | Women | 17 (33%) | 11.6 (3.3-37.0) |  |  |  |  |
| **Ethnicity** | Caucasian | 44 (85%) | 9.3 (6.8-54.7) | - | 0.1872 | - | 0.5456 |
|  | Other ethnicities | 8 (15%) | 8.6 (2.3-11.6) |  |  |  |  |
| **TMB^3^** | High | 18 (35%) | 37.0 (6.4-nr) | - | 0.0601 | - | 0.1938 |
|  | Low or intermediate | 34 (65%) | 7.9 (4.6-15.0) |  |  |  |  |
| **Type of immunotherapy** | Anti-PD-1/PD-L1 alone | 11 (21%) | 7.9 (3.3-15.0) | - | 0.4036 | - | - |
|  | Other immunotherapy regimens^4^ | 41 (79%) | 9.3 (6.4-54.7) |  |  |  |  |
| **UV^5^** | High | 24 (46%) | 8.9 (3.3-15.0) | **0.4 [0.2-1.0]** | **0.0329** | **0.4 [0.2-1.0]** | **0.0389** |
|  | Low | 28 (54%) | 37.0 (1.4-9.3) |  |  |  |  |

^1^Calculated using the log-rank test.

^2^Variables presenting a p-value ≤0.2 in univariate analysis were included in the multivariate model.

^3^TMB low = 1- 5 mutations/megabase; TMB intermediate = 6-19 mutations/megabase; TMB high ≥ 20 mutations/megabase

^4^Other immunotherapy: OX40 (n=3), anti-CD73 (n=1), anti-CTLA4 (n=15), OX40+anti-PD-1 (n=1), anti-PD-1+anti-CTLA4 (n=17), IDO+anti-PD-1 (n=1), high dose IL-2 (n=8), others (n=4).

^5^UV low < 0.7917 and UV high ≥ 0.7917 (as determined by the UV mutation signature enrichment UVMSE (Roberts *et al.*, 2013)).

**Abbreviations**: 95% CI = 95% confidence interval; CR = complete response; N = number; n.s. = non-significant; PFS = progression-free survival; PD-1 = programmed death receptor-1; PD-L1 programmed death receptor-ligand 1; TMB = tumor mutational burden; UV = ultraviolet.

## Supplemental Table 6: Factors associated with OS on immunotherapy for 99 non-melanoma patients treated with immunotherapy

No factor is significantly associated with overall survival in either univariate or multivariate analysis for non-melanoma patients treated with immunotherapy.

| **Variable** | **Group** | **Non-melanoma patients** | **OS (months)** | **Univariate^1^** | | **Multivariate^2^** | |
| --- | --- | --- | --- | --- | --- | --- | --- |
|  |  | **N = 99 (100%)** | **Median (range)** | **Hazard Ratio**  **[95% CI]** | **P-value** | **Hazard Ratio**  **[95% CI]** | **P-value** |
| **Age** | ≤60 years | 47 (47%) | 11.1 (4.7-21.0) | - | 0.6538 | - | - |
|  | >60 years | 52 (53%) | 11.2 (6.2-25.4) |  |  |  |  |
| **Gender** | Men | 58 (59%) | 11.2 (7.1-21.0) | - | 0.8224 | - | - |
|  | Women | 41 (41%) | nr (4.4-nr) |  |  |  |  |
| **Ethnicity** | Caucasian | 67 (68%) | 11.2 (7.1-25.4) | - | 0.4722 | - | - |
|  | Other ethnicities | 32 (32%) | 15.7 (2.8-nr) |  |  |  |  |
| **TMB^3^** | High | 20 (92%) | nr (11.2-nr) | - | 0.0571 | - | 0.5113 |
|  | Low or intermediate | 79 (92%) | 8.0 (5.1-21.0) |  |  |  |  |
| **Type of immunotherapy** | Anti-PD-1/PD-L1 alone | 91 (92%) | 11.2 (7.1-nr) | - | 0.6750 | - | - |
|  | Other immunotherapy regimens^4^ | 8 (8%) | 25.4 (1.8-25.4) |  |  |  |  |
| **UV^5^** | High | 22 (22%) | nr (7.6-nr) | - | 0.1777 | - | 0.8659 |
|  | Low | 77 (78%) | 11.1 (5.1-21.0) |  |  |  |  |

^1^Calculated using the log-rank test.

^2^Variables presenting a p-value ≤0.2 in univariate analysis were included in the multivariate model.

^3^TMB low = 1- 5 mutations/megabase; TMB intermediate = 6-19 mutations/megabase; TMB high ≥ 20 mutations/megabase

^4^Other immunotherapy: OX40 (n=3), anti-CD73 (n=1), anti-CTLA4 (n=15), OX40+anti-PD-1 (n=1), anti-PD-1+anti-CTLA4 (n=17), IDO+anti-PD-1 (n=1), high dose IL-2 (n=8), others (n=4).

^5^UV low < 0.7917 and UV high ≥ 0.7917 (as determined by the UV mutation signature enrichment UVMSE (Roberts *et al.*, 2013)).

**Abbreviations**: 95% CI = 95% confidence interval; N = number; n.s. = non-significant; OS = overall survival; PD-1 = programmed death receptor-1; PD-L1 programmed death receptor-ligand 1; TMB = tumor mutational burden; UV = ultraviolet.

## Supplemental Table 7: Factors associated with OS on immunotherapy for 52 melanoma patients treated with immunotherapy

No factor is significantly associated with overall survival in either univariate or multivariate analysis for melanoma patients treated with immunotherapy.

| **Variable** | **Group** | **Melanoma patients** | **OS (months)** | **Univariate^1^** | | **Multivariate^2^** | |
| --- | --- | --- | --- | --- | --- | --- | --- |
|  |  | **N = 52 (100%)** | **Median (range)** | **Hazard Ratio**  **[95% CI]** | **P-value** | **Hazard Ratio**  **[95% CI]** | **P-value** |
| **Age** | ≤60 years | 31 (60%) | 38.3 (16.3-nr) | - | 0.3259 | - | - |
|  | >60 years | 21 (40%) | nr (24.0-nr) |  |  |  |  |
| **Gender** | Men | 35 (67%) | nr (28.4-nr) | - | 0.5985 | - | - |
|  | Women | 17 (33%) | 38.3 (15.6-nr) |  |  |  |  |
| **Ethnicity** | Caucasian | 44 (85%) | nr (nr-nr) | - | 0.7463 | - | - |
|  | Other ethnicities | 8 (15%) | 38.3 (15.6-38.3) |  |  |  |  |
| **TMB^4^** | High | 18 (35%) | nr (nr-nr) | - | 0.1490 | - | - |
|  | Low or intermediate | 34 (65%) | 34.6 (24.0-nr) |  |  |  |  |
| **Type of immunotherapy** | Anti-PD-1/PD-L1 alone | 11 (21%) | nr (6.5-nr) | - | 0.6360 | - | - |
|  | Other immunotherapy regimens^5^ | 41 (79%) | 38.3 (28.4-nr) |  |  |  |  |
| **UV^6^** | High | 24 (46%) | 38.3 (24.0-nr) | - | 0.5076 | - | - |
|  | Low | 28 (54%) | nr (nr-nr) |  |  |  |  |

^1^Calculated using the log-rank test.

^2^Variables presenting a p-value ≤0.2 in univariate analysis were included in the multivariate model.

^3^TMB low = 1- 5 mutations/megabase; TMB intermediate = 6-19 mutations/megabase; TMB high ≥ 20 mutations/megabase

^4^Other immunotherapy: OX40 (n=3), anti-CD73 (n=1), anti-CTLA4 (n=15), OX40+anti-PD-1 (n=1), anti-PD-1+anti-CTLA4 (n=17), IDO+anti-PD-1 (n=1), high dose IL-2 (n=8), others (n=4).

^5^UV low < 0.7917 and UV high ≥ 0.7917 (as determined by the UV mutation signature enrichment UVMSE (Roberts *et al.*, 2013)).

**Abbreviations**: 95% CI = 95% confidence interval; N = number; n.s. = non-significant; OS = overall survival; PD-1 = programmed death receptor-1; PD-L1 programmed death receptor-ligand 1; TMB = tumor mutational burden; UV = ultraviolet.

**Supplemental Table 8: Univariate and multivariate analysis of factors affecting response rate, progression-free and overall survival for all patients treated with immunotherapy agents (N = 151)**

Melanoma tumor type and TMB-high are the only independent variables predicting response rate, PFS and OS (all p<0.03) in multivariate analysis of the entire cohort.

| **Factors associated with response rate on immunotherapy** | | | | | | | | | | | |
| --- | --- | --- | --- | --- | --- | --- | --- | --- | --- | --- | --- |
| **Variable** | **Group** | **All patients** | **CR/PR** | **SD/PD** | **Univariate^1^** | | | | **Multivariate^2^** | | |
|  |  | **N = 151 (100%)** | **N = 45 (30%)** | **N = 106 (70%)** | **Odds Ratio**  **[95% CI]** | | | **P-value** | **Odds Ratio [95% CI]** | | **P-value** |
| **Age** | ≤60 years (reference group) | 78 (52%) | 20 (26%) | 58 (74%) | 1.5 [0.7 - 3.0] | | | 0.2873 | - | | - |
|  | >60 years | 73 (48%) | 25 (34%) | 48 (66%) |  |  |  |  |  |  |  |
| **Gender** | Men | 93 (62%) | 33 (35%) | 60 (65%) | 2.1 [1.0-4.5] | | | 0.0675 | - | | - |
|  | Women (reference group) | 58 (38%) | 12 (21%) | 46 (79%) |  |  |  |  |  |  |  |
| **Ethnicity** | Caucasian | 111 (74%) | 38 (34%) | 73 (66%) | 2.5 [1.0-6.1] | | | 0.0685 | - | | 0.4401 |
|  | Other ethnicities (reference group) | 40 (26%) | 7 (17%) | 33 (83%) |  |  |  |  |  |  |  |
| **Tumor type** | Melanoma | 52 (34%) | 26 (50%) | 26 (50%) | **4.2 [2.0-8.8]** | | | **0.0001** | **3.9 [1.8-8.5]** | | **0.0007** |
|  | Other tumors^3^ (reference group) | 99 (66%) | 19 (19%) | 80 (81%) |  |  |  |  |  |  |  |
| **TMB^4^** | High | 38 (25%) | 22 (58%) | 16 (42%) | **5.4 [2.4-11.9]** | | | **<0.0001** | **5.0 [2.2-11.4]** | | **0.0002** |
|  | Low or intermediate (reference group) | 113 (75%) | 23 (20%) | 90 (80%) |  |  |  |  |  |  |  |
| **Type of immunotherapy** | Anti-PD-1/PD-L1 alone | 102 (68%) | 21 (21%) | 81 (79%) | **0.3 [0.1-0.6]** | | | **0.0006** | - | | 0.2773 |
|  | Other regimens^5^ (reference group) | 49 (32%) | 24 (49%) | 25 (51%) |  |  |  |  |  |  |  |
| **UV^6^** | High | 46 (30%) | 24 (52%) | 22 (48%) | **4.4 [2.1-9.2]** | | | **0.0002** | - | | 0.0636 |
|  | Low (reference group) | 105 (70%) | 21 (20%) | 84 (80%) |  |  |  |  |  |  |  |
| **Factors associated with PFS on immunotherapy** | | | | | | | | | | | |
| **Variable** | **Group** | **All patients** | **PFS (months)** | **Univariate^1^** | | | **Multivariate^2^** | | | | |
|  |  | **N = 151 (100%)** | **Median (range)** | **Hazard Ratio**  **[95% CI]** | | **P-value** | **Hazard Ratio**  **[95% CI]** | | | **P-value** | |
| **Age** | ≤60 years  (reference group) | 78 (52%) | 4.0 (3.2-5.9) | - | | 0.5396 | - | | | - | |
|  | >60 years | 73 (48%) | 5.5 (2.6-8.8) |  |  |  |  |  |  |  |  |
| **Gender** | Men | 93 (62%) | 5.8 (3.5-7.9) | - | | 0.0572 | - | | | 0.1752 | |
|  | Women  (reference group) | 58 (38%) | 3.6 (2.6-5.6) |  |  |  |  |  |  |  |  |
| **Ethnicity** | Caucasian | 111 (74%) | 5.9 (3.9-7.9) | **0.6 [0.4-0.8]** | | **0.0065** | - | | | 0.6837 | |
|  | Other ethnicities (reference group) | 40 (26%) | 2.6 (2.0-3.6) |  |  |  |  |  |  |  |  |
| **Tumor type** | Melanoma | 52 (34%) | 9.3 (6.8-37.0) | **0.3 [0.2-0.5]** | | **<0.0001** | **0.3 [0.2-0.5]** | | | **<0.0001** | |
|  | Other tumors^3^ (reference group) | 99 (66%) | 2.9 (2.0-3.8) |  |  |  |  |  |  |  |  |
| **TMB^4^** | High | 38 (25%) | 12.8 (6.5-n.r.) | **0.3 [0.2-0.5]** | | **<0.0001** | **0.3 [0.2-0.6]** | | | **<0.0001** | |
|  | Low or intermediate (reference group) | 113 (75%) | 3.3 (2.4-4.5) |  |  |  |  |  |  |  |  |
| **Type of immunotherapy** | Anti-PD-1/PD-L1 alone | 102 (68%) | 3.3 (2.1-4.5) | **2.6 [1.6-4.0]** | | **<0.0001** | - | | | 0.6541 | |
|  | Other immunotherapy regimens^5^  (reference group) | 49 (32%) | 9.3 (5.5-37.0) |  |  |  |  |  |  |  |  |
| **UV^6^** | High | 46 (30%) | 9.3 (6.4-n.r.) | **0.4 [0.3-0.7]** | | **0.0001** | - | | | 0.2716 | |
|  | Low  (reference group) | 105 (70%) | 3.2 (2.2-4.5) |  |  |  |  |  |  |  |  |
| **Factors associated with OS on immunotherapy** | | | | | | | | | | | |
| **Variable** | **Group** | **All patients** | **OS (months)** | **Univariate^1^** | | | **Multivariate^2^** | | | | |
|  |  | **N = 151 (100%)** | **Median (range)** | **Hazard Ratio**  **[95% CI]** | | **P-value** | **Hazard Ratio**  **[95% CI]** | | | **P-value** | |
| **Age** | ≤60 years | 78 (52%) | 25.4 (11.2-nr) | - | | 0.9165 | - | | | - | |
|  | >60 years | 73 (48%) | 28.4 (15.6-nr) |  |  |  |  |  |  |  |  |
| **Gender** | Men | 93 (62%) | 28.4 (15.7-nr) | - | | 0.6204 | - | | | - | |
|  | Women | 58 (38%) | 16.3 (15.6-nr) |  |  |  |  |  |  |  |  |
| **Ethnicity** | Caucasian | 111 (74%) | 28.4 (16.3-nr) | - | | 0.1192 | - | | | 0.8986 | |
|  | Other ethnicities | 40 (26%) | 15.6 (6.2-38.3) |  |  |  |  |  |  |  |  |
| **Tumor type** | Melanoma | 52 (34%) | nr (28.4-nr) | **0.2 [0.1-0.4]** | | **<0.0001** | **0.2 [0.1-0.4]** | | | **<0.0001** | |
|  | Other tumors^3^ | 99 (66%) | 11.2 (7.1-25.4) |  |  |  |  |  |  |  |  |
| **TMB^4^** | High | 38 (25%) | nr (nr-nr) | **0.3 [0.1-0.7]** | | **0.0036** | **0.4 [0.2-0.9]** | | | **0.0226** | |
|  | Low or intermediate | 113 (75%) | 16.3 (11.1-28.4) |  |  |  |  |  |  |  |  |
| **Type of immunotherapy** | Anti-PD-1/PD-L1 alone | 102 (68%) | 15.7 (7.6-nr) | **3.1 [1.6-5.9]** | | **0.0005** | - | | | 0.6969 | |
|  | Other immunotherapy regimens^5^ | 49 (32%) | 38.3 (25.4-nr) |  |  |  |  |  |  |  |  |
| **UV^6^** | High | 46 (30%) | nr (16.3-nr) | **0.4 [0.2-0.9]** | | **0.0139** | - | | | 0.5048 | |
|  | Low | 105 (70%) | 21.0 (11.1-34.6) |  |  |  |  |  |  |  |  |

^1^Calculated using Fisher's exact test.

^2^Variables presenting a p-value ≤0.2 in univariate analysis were included in the multivariate model.

^3^Tumors included: Adrenal carcinoma (n=1), appendix adenocarcinoma (n=1), basal cell carcinoma (n=2), bladder transitional cell carcinoma (n=4), breast cancer (n=3), cervical cancer (n=2), colorectal adenocarcinoma (n=5), cutaneous squamous cell carcinoma (n=8), hepatocellular carcinoma (n=3), head and neck (n=13), Merkel cell carcinoma (n=2), non-small cell lung carcinoma (n=36), ovarian carcinoma (n=2), pleural mesothelioma (n=1), prostate cancer (n=1), renal cell carcinoma (n=6), sarcoma (n=3), thyroid cancer (n=3), unknown primary squamous cell carcinoma (n=2), and urethral squamous cell carcinoma (n=1)

^4^TMB low = 1- 5 mutations/megabase; TMB intermediate = 6-19 mutations/megabase; TMB high ≥ 20 mutations/megabase

^5^Other immunotherapy: OX40 (n=3), anti-CD73 (n=1), anti-CTLA4 (n=15), OX40+anti-PD-1 (n=1), anti-PD-1+anti-CTLA4 (n=17), IDO+anti-PD-1 (n=1), high dose IL-2 (n=8), others (n=4).

^6^UV low < 0.7917 and UV high ≥ 0.7917 (as determined by the UV mutation signature enrichment UVMSE (Roberts *et al.*, 2013)).

**Abbreviations**: 95% CI = 95% confidence interval; CR = complete response; N = number; n.s. = non-significant; OS = overall survival; PD-1 = programmed death receptor-1; PD-L1 programmed death receptor-ligand 1; PD = progressive disease: PR = partial response; SD = stable disease; TMB = tumor mutational burden; UV = ultraviolet.

**Supplemental Table 9: Factors associated with response to immunotherapy for the treated 151 patients separated into higher and lower TMB groups.**

UVMSE is significantly different between responders and non-responders only in the low or intermediate TMB group (p = 0.0026). The p-value was calculated with Fisher’s exact test.

|  | **Low or Intermediate TMB^1^ (N=113)** | | **High TMB^1^**  **(N=38)** | |
| --- | --- | --- | --- | --- |
|  | **CR/PR**  **(N=23)** | **SD/PD**  **(N=90)** | **CR/PR**  **(N=22)** | **SD/PD**  **(N=16)** |
| **UV High^2^**  **(n=22)** | 10 (45%) | 12 (55%) | 14 (58%) | 10 (42%) |
| **UV Low^2^**  **(n=91)** | 13 (14%) | 78 (86%) | 8 (57%) | 6 (43%) |
| **p-value** | ***0.0026*** | | *1.0000* | |
| **Odds Ratio** [95% CI] | 5.0 [1.8 – 13.9] | | 1.1 [0.3 – 4.0] | |
| **Sensitivity** **of High UV in predicting responders** [95% CI] | 43% [23% – 66%] | | 64% [41% – 83%] | |
| **Specificity of High UV in predicting responders** [95% CI] | 87% [78% – 93%] | | 38% [15% – 65%] | |
| **Positive Predictive Value** [95% CI] | 45% [24% – 68%] | | 58% [37% – 78%] | |
| **Negative Predictive Value** [95% CI] | 86% [77% – 92%] | | 43% [18% – 71%] | |

^1^TMB low = 1- 5 mutations/megabase; TMB intermediate = 6-19 mutations/megabase; TMB high ≥ 20 mutations/megabase

^2^ UV low < 0.7917 and UV high ≥ 0.7917 (as determined by the UV mutation signature enrichment UVMSE (Roberts *et al.*, 2013)).

**Abbreviations**: 95% CI = 95% confidence interval; CR = complete response; N = number; PD = progressive disease: PR = partial response; SD = stable disease; TMB = tumor mutational burden; UV = ultraviolet.

## Supplemental Table 10: Univariate and multivariate analysis of factors affecting response rate for low/intermediate TMB patients treated with immunotherapy agents (N = 113)

In patients with low or intermediate TMB treated with immunotherapy, UVMSE was associated with clinical response in univariate (p = 0.0026) and in multivariate (p = 0.0108) analysis. Tumor type is also significant in both univariate (p = 0.0041) and multivariate analysis (p = 0.0139), but immunotherapy type was only significant in univariate analysis (p = 0.0101).

| **Variable** | **Group** | **Low/Intermediate^3^ TMB patients** | **CR/PR** | **SD/PD** | **Univariate^1^** | | **Multivariate^2^** | |
| --- | --- | --- | --- | --- | --- | --- | --- | --- |
|  |  | **N = 113 (100%)** | **N = 23 (20%)** | **N = 90 (80%)** | **Odds Ratio**  **[95% CI]** | **P-value** | **Odds Ratio**  **[95% CI]** | **P-value** |
| **Age** | ≤60 years (reference group) | 67 (59%) | 15 (22%) | 52 (78%) | 0.7 [0.3 - 1.9] | 0.6364 | - | - |
|  | >60 years | 46 (41%) | 8 (17%) | 38 (83%) |  |  |  |  |
| **Gender** | Men | 64 (57%) | 15 (23%) | 49 (77%) | 1.6 [0.6 - 4.0] | 0.4802 | - | - |
|  | Women  (reference group) | 49 (43%) | 8 (16%) | 41 (84%) |  |  |  |  |
| **Ethnicity** | Caucasian | 77 (68%) | 18 (23%) | 59 (77%) | 1.9 [0.6 - 5.6] | 0.3192 | - | - |
|  | Other ethnicities (reference group) | 36 (32%) | 5 (14%) | 31 (86%) |  |  |  |  |
| **Tumor type** | Melanoma | 34 (30%) | 13 (38%) | 21 (62%) | **4.3 [1.7 - 11.1]** | **0.0041** | **3.5 [1.3 - 9.5]** | **0.0139** |
|  | Other tumors^4^ (reference group) | 79 (70%) | 10 (13%) | 69 (87%) |  |  |  |  |
| **Type of immunotherapy** | Anti-PD-1/PD-L1 alone | 80 (21%) | 11 (14%) | 69 (86%) | **0.3 [0.1 - 0.7]** | **0.0101** | - | 0.4392 |
|  | Other regimens^5^ (reference group) | 33 (79%) | 12 (36%) | 21 (84%) |  |  |  |  |
| **UV^6^** | High | 22 (19%) | 10 (45%) | 12 (55%) | **5.0 [1.8 - 13.9]** | **0.0026** | **4.0 [1.4 - 11.7]** | **0.0108** |
|  | Low  (reference group) | 91 (81%) | 13 (14%) | 78 (86%) |  |  |  |  |

^1^Calculated using Fisher's exact test.

^2^Variables presenting a p-value ≤0.2 in univariate analysis were included in the multivariate model.

^3^TMB low = 1- 5 mutations/megabase; TMB intermediate = 6-19 mutations/megabase; TMB high ≥ 20 mutations/megabase

^4^Tumors included: Adrenal carcinoma (n=1), appendix adenocarcinoma (n=1), basal cell carcinoma (n=2), bladder transitional cell carcinoma (n=4), breast cancer (n=3), cervical cancer (n=2), colorectal adenocarcinoma (n=5), cutaneous squamous cell carcinoma (n=8), hepatocellular carcinoma (n=3), head and neck (n=13), Merkel cell carcinoma (n=2), non-small cell lung carcinoma (n=36), ovarian carcinoma (n=2), pleural mesothelioma (n=1), prostate cancer (n=1), renal cell carcinoma (n=6), sarcoma (n=3), thyroid cancer (n=3), unknown primary squamous cell carcinoma (n=2), and urethral squamous cell carcinoma (n=1)

^5^Other immunotherapy: OX40 (n=3), anti-CD73 (n=1), anti-CTLA4 (n=15), OX40+anti-PD-1 (n=1), anti-PD-1+anti-CTLA4 (n=17), IDO+anti-PD-1 (n=1), high dose IL-2 (n=8), others (n=4).

^6^UV low < 0.7917 and UV high ≥ 0.7917 (as determined by the UV mutation signature enrichment UVMSE (Roberts *et al.*, 2013)).

**Abbreviations**: 95% CI = 95% confidence interval; CR = complete response; N = number; n.s. = non-significant; PD-1 = programmed death receptor-1; PD-L1 programmed death receptor-ligand 1; PD = progressive disease: PR = partial response; SD = stable disease; TMB = tumor mutational burden; UV = ultraviolet; UVMSE = UV Mutational Signature Enrichment.

## Supplemental Table 11: Factors associated with PFS on immunotherapy for 113 low/intermediate TMB patients treated with immunotherapy

UVMSE (p = 0.0359), immunotherapy type (p = 0.0004), and tumor type (p < 0.0001) are independent factors predicting PFS in low and intermediate TMB patients treated with immunotherapy in only univariate analysis. Only tumor type is statistically significant in multivariate analysis (p < 0.0001).

| **Variable** | **Group** | **Low/Intermediate^3^ TMB patients** | **PFS (months)** | **Univariate^1^** | | **Multivariate^2^** | |
| --- | --- | --- | --- | --- | --- | --- | --- |
|  |  | **N = 113 (100%)** | **Median (range)** | **Hazard Ratio**  **[95% CI]** | **P-value** | **Hazard Ratio**  **[95% CI]** | **P-value** |
| **Age** | ≤60 years  (reference group) | 67 (60%) | 3.9 (2.6-5.0) | 1.3 [0.9 - 2.0] | 0.1978 | - | 0.9358 |
|  | >60 years | 46 (40%) | 2.3 (1.8-5.6) |  |  |  |  |
| **Gender** | Men | 64 (57%) | 3.5 (2.2-5.8) | 0.8 [0.5 - 1.3] | 0.2818 | - | - |
|  | Women  (reference group) | 49 (43%) | 3.3 (2.0-4.2) |  |  |  |  |
| **Ethnicity** | Caucasian | 77 (68%) | 3.9 (2.2-5.9) | 0.8 [0.5 - 1.2] | 0.1872 | - | 0.7002 |
|  | Other ethnicities  (reference group) | 36 (32%) | 2.6 (2.0-3.6) |  |  |  |  |
| **Tumor Type** | Melanoma | 34 (30%) | 7.9 (4.6-15.0) | **0.3 [0.2 - 0.5]** | **< 0.0001** | **0.3 [0.2 - 0.5]** | **< 0.0001** |
|  | Other tumors^4^ (reference group) | 79 (70%) | 2.1 (1.8-3.1) |  |  |  |  |
| **Type of immunotherapy** | Anti-PD-1/PD-L1 alone | 80 (71%) | 2.2 (1.9-3.5) | **2.3 [1.4-3.8]** | **0.0004** | - | 0.9554 |
|  | Other immunotherapy regimens^5^  (reference group) | 33 (29%) | 5.8 (3.4-11.6) |  |  |  |  |
| **UV^6^** | High | 22 (19%) | 6.6 (3.5-9.3) | **0.6 [0.3 - 1.0]** | **0.0359** | **-** | 0.4320 |
|  | Low  (reference group) | 91 (81%) | 2.8 (2.1-3.9) |  |  |  |  |

^1^Calculated using Fisher's exact test.

^2^Variables presenting a p-value ≤0.2 in univariate analysis were included in the multivariate model.

^3^TMB low = 1- 5 mutations/megabase; TMB intermediate = 6-19 mutations/megabase; TMB high ≥ 20 mutations/megabase

^4^Tumors included: Adrenal carcinoma (n=1), appendix adenocarcinoma (n=1), basal cell carcinoma (n=2), bladder transitional cell carcinoma (n=4), breast cancer (n=3), cervical cancer (n=2), colorectal adenocarcinoma (n=5), cutaneous squamous cell carcinoma (n=8), hepatocellular carcinoma (n=3), head and neck (n=13), Merkel cell carcinoma (n=2), non-small cell lung carcinoma (n=36), ovarian carcinoma (n=2), pleural mesothelioma (n=1), prostate cancer (n=1), renal cell carcinoma (n=6), sarcoma (n=3), thyroid cancer (n=3), unknown primary squamous cell carcinoma (n=2), and urethral squamous cell carcinoma (n=1)

^5^Other immunotherapy: OX40 (n=3), anti-CD73 (n=1), anti-CTLA4 (n=15), OX40+anti-PD-1 (n=1), anti-PD-1+anti-CTLA4 (n=17), IDO+anti-PD-1 (n=1), high dose IL-2 (n=8), others (n=4).

^6^UV low < 0.7917 and UV high ≥ 0.7917 (as determined by the UV mutation signature enrichment UVMSE (Roberts *et al.*, 2013)).

**Abbreviations**: 95% CI = 95% confidence interval; CR = complete response; N = number; n.s. = non-significant; PFS = progression-free survival; PD-1 = programmed death receptor-1; PD-L1 programmed death receptor-ligand 1; TMB = tumor mutational burden; UV = ultraviolet.

## Supplemental Table 12: Factors associated with OS on immunotherapy for 113 low/intermediate TMB patients treated with immunotherapy

Immunotherapy type (p = 0.0036) and tumor type (p < 0.0001) are independent factors predicting OS in low and intermediate TMB patients treated with immunotherapy in univariate analysis. Only tumor type is statistically significant in multivariate analysis (p < 0.0001). UVMSE was not significant in either analysis.

| **Variable** | **Group** | **Low/Intermediate^3^ TMB patients** | **OS (months)** | **Univariate^1^** | | **Multivariate^2^** | |
| --- | --- | --- | --- | --- | --- | --- | --- |
|  |  | **N = 113 (100%)** | **Median (range)** | **Hazard Ratio**  **[95% CI]** | **P-value** | **Hazard Ratio**  **[95% CI]** | **P-value** |
| **Age** | ≤60 years  (reference group) | 67 (60%) | 21.0 (15.6-38.3) | 1.5 [0.9 - 2.7] | 0.1517 | - | 0.9992 |
|  | >60 years | 46 (40%) | 15.7 (5.1-25.4) |  |  |  |  |
| **Gender** | Men | 64 (57%) | 21.0 (8.0-34.6) | 0.8 [0.5 - 1.5] | 0.5199 | - | - |
|  | Women  (reference group) | 49 (43%) | 15.6 (7.4-38.3) |  |  |  |  |
| **Ethnicity** | Caucasian | 77 (68%) | 21.0 (8.2-28.4) | 0.9 [0.5 - 1.7] | 0.7651 | - | - |
|  | Other ethnicities  (reference group) | 36 (32%) | 15.6 (6.2-38.3) |  |  |  |  |
| **Tumor Type** | Melanoma | 34 (30%) | 34.6 (24.0-nr) | **0.2 [0.1 - 0.4]** | **< 0.0001** | **0.2 [0.1 - 0.4]** | **< 0.0001** |
|  | Other tumors^4^ (reference group) | 79 (70%) | 8.0 (5.1-21.0) |  |  |  |  |
| **Type of immunotherapy** | Anti-PD-1/PD-L1 alone | 80 (71%) | 11.1 (7.1-21.0) | **2.7 [1.4-5.4]** | **0.0036** | - | 0.9495 |
|  | Other immunotherapy regimens^5^  (reference group) | 33 (29%) | 28.4 (24.0-nr) |  |  |  |  |
| **UV^6^** | High | 22 (19%) | 6.6 (3.5-9.3) | 0.4 [0.2 - 1.0] | 0.0518 | **-** | 0.2943 |
|  | Low  (reference group) | 91 (81%) | 15.6 (8.0-25.4) |  |  |  |  |

^1^Calculated using Fisher's exact test.

^2^Variables presenting a p-value ≤0.2 in univariate analysis were included in the multivariate model.

^3^TMB low = 1- 5 mutations/megabase; TMB intermediate = 6-19 mutations/megabase; TMB high ≥ 20 mutations/megabase

^4^Tumors included: Adrenal carcinoma (n=1), appendix adenocarcinoma (n=1), basal cell carcinoma (n=2), bladder transitional cell carcinoma (n=4), breast cancer (n=3), cervical cancer (n=2), colorectal adenocarcinoma (n=5), cutaneous squamous cell carcinoma (n=8), hepatocellular carcinoma (n=3), head and neck (n=13), Merkel cell carcinoma (n=2), non-small cell lung carcinoma (n=36), ovarian carcinoma (n=2), pleural mesothelioma (n=1), prostate cancer (n=1), renal cell carcinoma (n=6), sarcoma (n=3), thyroid cancer (n=3), unknown primary squamous cell carcinoma (n=2), and urethral squamous cell carcinoma (n=1)

^5^Other immunotherapy: OX40 (n=3), anti-CD73 (n=1), anti-CTLA4 (n=15), OX40+anti-PD-1 (n=1), anti-PD-1+anti-CTLA4 (n=17), IDO+anti-PD-1 (n=1), high dose IL-2 (n=8), others (n=4).

^6^UV low < 0.7917 and UV high ≥ 0.7917 (as determined by the UV mutation signature enrichment UVMSE (Roberts *et al.*, 2013)).

**Abbreviations**: 95% CI = 95% confidence interval; CR = complete response; N = number; n.s. = non-significant; PFS = progression-free survival; PD-1 = programmed death receptor-1; PD-L1 programmed death receptor-ligand 1; TMB = tumor mutational burden; UV = ultraviolet.

## Supplemental Figure 1: UV signature enrichment analysis in a cohort of 151 patients and correlation to the response to immunotherapy. Patient data was not filtered based on genomic coordinates corresponding to the regions sequenced by Foundation Medicine as shown in Figure 4; all sequence data was analyzed for this figure.

The UVMSE calculated with all genomic regions from the cohort of 151 Moores Cancer Center patients was used to assess the validity of the UVMSE as a method of measuring degree of UV mutation in tumor samples. See **Figure 4** for the UVMSE calculated on the same cohort with only the Foundation Medicine genomic regions. Red lines = mean (95% CI); all p-values were calculated using the Mann-Whitney U-test.

Panel A: Comparison of UVMSE in melanoma versus non-melanoma diagnosed patients. Melanoma patients had an average UVMSE of 0.8275 (95% CI: 0.8082 - 0.8467) while non-melanoma patients had an average UVMSE of 0.7950 (95% CI: 0.7849 - 0.8050). The difference was significant with a p-value of 0.0002.

Panel B: A comparison of the average of all non-UVMSE values in melanoma vs non-melanoma diagnosed patients. This difference was not significant with a p-value of 0.0838, showing that UVMSE is useable as a specific measurement of mutation enrichment due to UV light exposure.

Panel C: UVMSE is able to differentiate between negative (SD or PD within 6 months) and positive (CR or PR within 6 months) PFS outcomes to immunotherapy in the entire 151-patient cohort. The average UVMSE of the positive outcome group was 0.8321 (95% CI: 0.8075 - 0.8567) while the average UVMSE of the negative outcome group was 0.7951 (95% CI: 0.7869 - 0.8034). This difference was statistically significant with a p-value of 0.0165.

Panel D: Within only the cohort of 52 melanoma patients, UVMSE is unable to distinguish between positive and negative outcomes (p = 0.1564). However, the positive outcome group still has a higher average UVMSE of 0.8436 (95% CI: 0.8111 - 0.8760) than the negative outcome group at 0.8113 (95% CI: 0.7901 - 0.8326).

**

**

## Supplemental Figure 2: UV signature enrichment analysis in a cohort of 328 acral and cutaneous melanomas



The UVMSE of the Cutaneous Melanoma cohort from TCGA (n=290) was compared with a cohort of acral melanomas from (Liang *et al.*, 2017) (n=38) in order to determine whether the UVMSE score is sufficiently sensitive to distinguish degrees of UV mutagenesis in TCGA samples. The acral melanomas were deemed to have low UV exposure while the SKCM ones were deemed to have high UV exposure. The average UVMSE of the acral/low group was 1.206 (95% CI: 0.8290 – 1.583) while the average UVMSE of the SKCM/high group was 1.842 (95% CI: 1.762 – 1.922). The difference between the 2 groups was significant (p < 0.0001). This shows that UVMSE is a sensitive and specific quantifier of UV mutagenesis. Red lines = Mean (95% CI); all p-values were calculated using the Mann-Whitney U-test.
